# Long noncoding RNA LINC00662 promotes M2 macrophage polarization and hepatocellular carcinoma progression via activating Wnt/β‐catenin signaling

**DOI:** 10.1002/1878-0261.12606

**Published:** 2019-12-21

**Authors:** Xiaohui Tian, Yuanyuan Wu, Yating Yang, Jiaxin Wang, Menglan Niu, Shanjun Gao, Tao Qin, Dengke Bao

**Affiliations:** ^1^ Department of Clinical Laboratory Henan Provincial People's Hospital Henan University People’s Hospital Zhengzhou China; ^2^ Laboratory of Cancer Biomarkers and Liquid Biopsy School of Pharmacy Henan University Kaifeng China; ^3^ Microbiome Laboratory Henan Provincial People's Hospital Henan University People’s Hospital Zhengzhou China; ^4^ Department of Hepatobiliary Pancreatic Surgery Henan Provincial People's Hospital Henan University People’s Hospital Zhengzhou China

**Keywords:** ceRNA, hepatocellular carcinoma, LINC00662, long noncoding RNA, macrophage polarization, Wnt/β‐catenin signaling

## Abstract

Tumor‐associated macrophages have important roles in hepatocellular carcinoma (HCC) initiation and progression. Long noncoding RNAs (lncRNAs) have also been reported to be involved in HCC. In this study, we explored how lncRNA LINC00662 may influence HCC progression through both tumor cell‐dependent and macrophage‐dependent mechanisms. LINC00662 was found to be upregulated in HCC, and high LINC00662 levels correlated with poor survival of HCC patients. LINC00662 upregulated WNT3A expression and secretion via competitively binding miR‐15a, miR‐16, and miR‐107. Through inducing WNT3A secretion, LINC00662 activated Wnt/β‐catenin signaling in HCC cells in an autocrine manner and further promoted HCC cell proliferation, cell cycle, and tumor cell invasion, while repressing HCC cell apoptosis. In addition, acting through WNT3A secretion, LINC00662 activated Wnt/β‐catenin signaling in macrophages in a paracrine manner and further promoted M2 macrophage polarization. Via activating Wnt/β‐catenin signaling and M2 macrophages polarization, LINC00662 significantly promoted HCC tumor growth and metastasis *in vivo*. Hence, targeting LINC00662 may provide novel therapeutic strategy against HCC.

## Graphical abstract

Schematic model of LINC00662 functions. LINC00662 upregulates WNT3A expression and secretion via competitively binding miR‐15a/16/107. Secreted WNT3A activates Wnt/β‐catenin in HCC cells in an autocrine manner and promotes HCC cell proliferation and invasion. Secreted WNT3A also activates Wnt/β‐catenin in macrophages in a paracrine manner and promotes M2 macrophage polarization. Finally, LINC00662 promotes HCC tumor growth and metastasis via upregulating WNT3A.

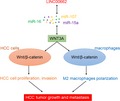

**Abbreviations**
ANOVA: analysis of variance
CCK‐8: cell counting kit‐8
ceRNA: competitive endogenous RNA
CM: conditioned media
EdU: 5‐ethynyl‐2'‐deoxyuridine
ELISA: enzyme‐linked immunosorbent assay
FBS: fetal bovine serum
HCC: hepatocellular carcinoma
HE: hematoxylin and eosin
IF: immunofluorescence
IHC: immunohistochemistry
lncRNA: long noncoding RNA
miRNA: microRNA
NC: negative control
PMA: phorbol 12‐myristate 13‐acetate
qRT/PCR: quantitative real‐time polymerase chain reaction
RIP: RNA immunoprecipitation
TAM: tumor‐associated macrophage
TME: tumor microenvironment

## 1 Introduction

According to global cancer statistics in 2018, liver cancer ranks sixth in incidence and third in mortality, which accounts for 841 080 estimated new cases and 781 631 estimated deaths in 2018 worldwide (Bray *et al.*, 2018). Hepatocellular carcinoma (HCC) accounts for the overwhelming majority of liver cancer (Forner *et al.*, 2012). Although great advances have been made in surgery, transcatheter arterial chemoembolization, molecular targeted therapy, and immunotherapy, the prognosis of most HCC patients is still very poor (Meyer *et al.*, 2017). Thus, it is urgently needed to develop more efficient therapeutic strategies against HCC (Xue *et al.*, 2019).

Most of previous studies have mainly focused on the regulation of behaviors of HCC cell, such as the viability and migration of HCC cells (Dhar *et al.*, 2018; Liu *et al.*, 2017; Zhu *et al.*, 2016). However, more and more studies have revealed that the tumor microenvironment (TME) has critical roles in the progression of HCC (Liu *et al.*, 2019a; Yang *et al.*, 2019). Furthermore, the crosstalk between HCC cells and TME components may dramatically drive HCC malignancy and resistance to therapy (Lu *et al.*, 2019; Yang *et al.*, 2018). Tumor‐associated macrophages (TAMs) are the major components of TME (Yin *et al.*, 2017). Increasing evidences have shown that TAMs are correlated with survival of HCC patients (Budhu *et al.*, 2006; Wan *et al.*, 2014). Under different stimulations, macrophages can be polarized into M1 macrophages or M2 macrophages. M1 macrophages have tumoricidal activity (Zhang *et al.*, 2018b). Conversely, M2 macrophages have protumor activity via promoting HCC cell proliferation, migration, angiogenesis, immunosuppressive microenvironment, and so on (Chen *et al.*, 2019b; Wang *et al.*, 2017; Zhang *et al.*, 2018a). Elucidating the molecular mechanisms underlying the interaction between HCC cells and macrophages may provide novel therapeutic strategies for HCC.

Genome and transcriptome sequencings have found that the number of protein‐coding genes is about 21 000, while the number of long noncoding RNAs (lncRNAs) is more than 58 000 (Iyer *et al.*, 2015). As a class of regulatory transcripts with limited protein‐coding potential, lncRNAs gradually show important roles in various pathophysiological processes (Esposito *et al.*, 2019b; Hu *et al.*, 2018; Li *et al.*, 2017). Previous studies have shown that lncRNAs are frequently deregulated in many cancers, including HCC (Berger *et al.*, 2018; Wang *et al.*, 2018). Furthermore, lncRNAs play important roles in HCC cell proliferation, cell cycle, apoptosis, senescence, migration, invasion, drug resistance, and so on (Liu *et al.*, 2016; Wang *et al.*, 2014; Yuan *et al.*, 2017). However, the roles of lncRNAs in the interaction between HCC cells and TME are mostly unknown (Chen *et al.*, 2019a). The functional mechanisms of lncRNAs are complex and various (Swier *et al.*, 2019; Yao *et al.*, 2019). One of the important mechanisms for cytoplasmic lncRNAs is to competitively bind common microRNAs (miRNAs) and relieve the repressive roles of common miRNAs on their targets (Cesana *et al.*, 2011; Yuan *et al.*, 2014). These lncRNAs were classed as competitive endogenous RNAs (ceRNAs) (Salmena *et al.*, 2011).

Wnt/β‐catenin signaling is evolutionarily conserved and required for embryonic development and tissue homeostasis (Petridou *et al.*, 2019). Wnt/β‐catenin signaling is also revealed to be involved in the initiation and development of many cancers (Esposito *et al.*, 2019a). Furthermore, Wnt/β‐catenin signaling has been shown to regulate differentiation of monocyte into macrophage (Ye *et al.*, 2019). Wnt ligands secreted by HCC cells activate Wnt/β‐catenin signaling in macrophage and induce M2 macrophage polarization (Yang *et al.*, 2018).

In this study, we identified a lncRNA LINC00662, which upregulates WNT3A expression and secretion via ceRNA mechanism. Through inducing WNT3A secretion, LINC00662 promotes HCC progression and M2 macrophage polarization.

## 2 Materials and methods

### 2.1 Cell culture and treatment

Immortalized hepatic normal cell QSG‐7701, HCC cells HCCLM3, MHCC97H, Huh7, and SK‐HEP‐1, and human monocytic THP‐1 cell were obtained from the Chinese Academy of Sciences Cell Bank. QSG‐7701 and THP‐1 were cultured in RPMI‐1640 medium (Gibco, Invitrogen, Carlsbad, CA, USA). HCCLM3 and Huh7 were cultured in Dulbecco’s modified Eagle’s medium (DMEM) (Gibco). MHCC97H and SK‐HEP‐1 were cultured in Eagle's minimum essential medium (MEM) (Gibco). All these media were routinely supplemented with 10% fetal bovine serum (FBS) (Hyclone, Logan, UT, USA). THP‐1 monocyte was treated with 150 nm phorbol 12‐myristate 13‐acetate (PMA) (Sigma‐Aldrich, St Louis, MO, USA) for 24 h to be differentiated into macrophage. To obtain conditioned media (CM) from HCC cells, indicated HCC cells with 80% confluence were washed three times with FBS‐free medium and maintained in fresh FBS‐free medium for another 72 h. Next, the supernatant was filtered through 0.22‐μm filter and collected as CM. THP‐1 differentiated macrophage was treated with the CM from HCC cells for another 48 h before RNA or protein extraction. Where indicated in this article, THP‐1 differentiated macrophage was treated with the CM from HCC cells added with 25 μm ICG‐001 (Selleck, Houston, TX, USA).

### 2.2 RNA extraction and quantitative real‐time polymerase chain reaction (qRT/PCR)

Total RNA was extracted from indicated tissues and cells using the Trizol reagent (Invitrogen). First‐strand cDNA was synthesized with the M‐MLV Reverse Transcriptase (Invitrogen). qRT/PCR was conducted using SYBR Green PCR Master Mix (TaKaRa, Dalian, China) on a StepOne Plus Real‐Time PCR System (Applied Biosystems, Foster City, CA, USA). The primer sequences for qRT/PCR are presented in Table S1. For miRNAs analysis, qRT/PCR was performed using TaqMan microRNA assays (Applied Biosystems). β‐Actin was used as an internal control. Relative quantification was calculated based on the comparative Ct method. For exact quantification of expression levels of lncRNA and miRNAs, the lncRNA expression vector and reverse‐transcribed miRNAs cDNA were used as standard templates to formulate standard curves with limit dilution approaches. The exact expression levels of lncRNA and miRNAs per cell were calculated according to cell counts and molecular weights.

### 2.3 Northern blot

Northern blot was conducted using the NorthernMax™ Kit (Ambion, Grand Island, NY, USA). A total of 10 µg RNA from indicated cells was subjected to formaldehyde gel electrophoresis and transferred to a Biodyne Nylon membrane (Pall, Port Washington, NY, USA). Biotin‐16‐dUTP (Roche, Mannheim, Germany)‐labeled LINC00662 cDNA probe was acquired with PCR. The primer sequences were presented in Table S1. After 60 min of prehybridization in ULTRAhyb™‐Oligo buffer (Ambion), the membrane was hybridized at 68 °C in ULTRAhyb™‐Oligo buffer containing the denatured probe for 12 h. After washes following the provided protocol, the membrane was detected with an Odyssey infrared scanner (Li‐Cor, Lincoln, NE, USA).

### 2.4 RNA fluorescence *in situ* hybridization

For *in situ* detection of LINC00662 in HCC cells, the LINC00662 probes were designed and synthesized by Advanced Cell Diagnostics (ACD, Newark, CA, USA). The hybridization and fluorescence detection were conducted with the RNAscope Fluorescent Multiplex Detection Kit (ACD). The localization of LINC00662 in HCC cells was observed using confocal laser scanning microscopy (Leica, Wetzlar, Germany).

### 2.5 Isolation of cytoplasmic and nuclear RNA

Cytoplasmic and nuclear RNA were extracted with the PARIS Kit (Ambion) following the provided protocol. The level of LINC00662 in the cytoplasmic and nuclear RNA was detected by qRT/PCR described above.

### 2.6 Vectors construction

The cDNA encoding LINC00662 was PCR‐amplified with Thermo Scientific Phusion Flash High‐Fidelity PCR Master Mix (Thermo Fisher, Waltham, MA, USA) and subcloned into the Kpn I and EcoR I sites of pcDNA™3.1(+) vector (Invitrogen), termed as pcDNA3.1‐LINC00662. The primer sequences are presented in Table S1. pcDNA3.1‐LINC00662 with mutations in miR‐15a/16/107 binding sites was produced by GenScript (Nanjing, China), termed as pcDNA3.1‐LINC‐mut. pcDNA3.1‐LINC00662 and pcDNA3.1‐LINC‐mut were double‐digested using Kpn I and EcoR I, and the lncRNA coding sequences were inserted into pSPT19 (Roche), termed as pSPT19‐LINC00662 and pSPT19‐LINC‐mut, respectively. In addition, pcDNA3.1‐LINC00662 and pcDNA3.1‐LINC‐mut were double‐digested using Nhe I and Not I, and the lncRNA coding sequences were inserted into pSL‐MS2‐12X (Addgene, Watertown, MA, USA), termed as pSL‐MS2‐LINC00662 and pSL‐MS2‐LINC‐mut, respectively. The oligonucleotides for shRNAs targeting LINC00662 were produced and inserted into the shRNA expression vector pGPU6/GFP/Neo (GenePharma, Shanghai, China), termed as shRNA‐LINC‐1 and shRNA‐LINC‐2. The shRNA sequences are presented in Table S1. The LINC00662 sequences containing miR‐15a/16/107 binding sites were PCR‐amplified with Thermo Scientific Phusion Flash High‐Fidelity PCR Master Mix (Thermo Fisher) and subcloned into the Sac I and Xho I sites of pmirGLO vector (Promega, Madison, WI, USA). pcDNA3.1‐LINC00662 and pcDNA3.1‐LINC‐mut were used as template, and therefore, the constructed vectors were named as pmirGLO‐LINC00662 and pmirGLO‐LINC00662‐mut, respectively. 3' untranslated region (UTR) of WNT3A containing miR‐15a/16/107 binding sites was PCR‐amplified with Thermo Scientific Phusion Flash High‐Fidelity PCR Master Mix (Thermo Fisher) and subcloned into the Sac I and Xho I sites of pmirGLO vector (Promega), termed as pmirGLO‐WNT3A.

### 2.7 Stable cell line construction

To obtain LINC00662 stably overexpressed HCC cells, pcDNA3.1, pcDNA3.1‐LINC00662, and pcDNA3.1‐LINC‐mut were transfected into HCCLM3 and MHCC97H cells using Lipofectamine 3000 (Invitrogen) following the provided protocol. To obtain LINC00662 stably silenced HCC cells, shRNA‐NC, shRNA‐LINC‐1, and shRNA‐LINC‐2 were transfected into Huh7 and SK‐HEP‐1 cells using Lipofectamine 3000 (Invitrogen). Forty‐eight hours after transfection, the cells were selected with neomycin (800 µg·mL^−1^) for 4 weeks. The overexpression and silencing efficiencies of LINC00662 were detected by qRT/PCR.

### 2.8 Luciferase reporter assay

pmirGLO, pmirGLO‐LINC00662, or pmirGLO‐LINC00662‐mut were cotransfected with miR‐15a mimics, miR‐16 mimics, miR‐107 mimics, miR‐NC (negative control of miRNA mimics), miR‐15a inhibitors, miR‐16 inhibitors, miR‐107 inhibitors, or inh‐NC (negative control of miRNA inhibitors) into HCCLM3 cells using Lipofectamine 3000. pmirGLO‐WNT3A was cotransfected with pcDNA3.1, pcDNA3.1‐LINC00662, or pcDNA3.1‐LINC‐mut into HCCLM3 cells using Lipofectamine 3000. pmirGLO‐WNT3A was cotransfected with shRNA‐NC, shRNA‐LINC‐1, or shRNA‐LINC‐2 into SK‐HEP‐1 cells using Lipofectamine 3000. Forty‐eight hours later, the luciferase activities were detected by the Dual‐Luciferase Reporter Assay System (Promega) following the provided protocol.

### 2.9 RNA pull‐down

Wild‐type and miR‐15a/16/107 binding sites mutated LINC00662 were *in vitro*‐transcribed from pSPT19‐LINC00662 and pSPT19‐LINC‐mut, respectively, and biotin‐labeled using the Biotin RNA Labeling Mix (Roche) and T7 RNA polymerase (Roche). After being treated with RNase‐free DNase I (Roche) and purified by a RNeasy Mini Kit (Qiagen, Valencia, CA, USA), 3 µg of *in vitro*‐transcribed LINC00662 or LINC00662‐mut was incubated with 1 mg of whole‐cell lysates from HCCLM3 cells for 1 h at 25 °C. Next, the complexes were isolated by streptavidin agarose beads (Invitrogen). The RNA present in the pull‐down material was measured by qRT/PCR.

### 2.10 RNA immunoprecipitation (RIP)

pSL‐MS2‐12X, pSL‐MS2‐LINC00662, or pSL‐MS2‐LINC‐mut was cotransfected with pMS2‐GFP (Addgene) into HCCLM3 cells using Lipofectamine 3000. Forty‐eight hours later, RNA immunoprecipitation (RIP) experiments were conducted with the Magna RIP™ RNA‐Binding Protein Immunoprecipitation Kit (Millipore, Bedford, MA, USA) and a primary GFP antibody (Roche). The enriched RNA was measured by qRT/PCR.

### 2.11 Western blot

Total cell extracts from indicated cells were obtained using the radioimmunoprecipitation assay (RIPA) lysis buffer (Beyotime, Shanghai, China). Protein concentration was determined by the Bicinchoninic acid (BCA) kit (Beyotime). After being separated by sodium dodecyl sulfate/polyacrylamide gel electrophoresis, the protein was transferred onto nitrocellulose membrane. After being blocked by 5% bovine serum albumin (BSA) for 2 h at room temperature, the membrane was incubated with primary antibodies against WNT3A (ab81614, 1 : 1000, Abcam, Cambridge, MA, USA), β‐catenin (ab32572, 1 : 2500, Abcam), or β‐actin (T0022, 1 : 10 000, Affinity, Changzhou, China) overnight at 4 °C. After three rinses, the membrane was incubated with goat anti‐mouse IgG H&L (IRDye^®^ 680RD) preadsorbed (ab216776, 1 : 10 000, Abcam) or goat anti‐rabbit IgG H&L (IRDye^®^ 800CW) preadsorbed (ab216773, 1 : 10 000, Abcam) for 1 h at room temperature. Finally, the membrane was detected with an Odyssey infrared scanner (Li‐Cor, Lincoln, NE, USA).

### 2.12 Enzyme‐linked immunosorbent assay (ELISA)

Indicated HCC cells with 80% confluence were washed three times with FBS‐free medium and maintained in fresh FBS‐free medium for another 48 h. Then, the supernatant was collected and the concentration of WNT3A in the supernatant was measured by a human WNT3A ELISA Kit (MyBioSource, San Diego, CA, USA).

### 2.13 Cell proliferation assay

Cell proliferation was detected by Cell Counting Kit‐8 (CCK‐8) and 5‐ethynyl‐2'‐deoxyuridine (EdU) incorporation experiments. In CCK‐8 experiments, 3000 HCC cells per well were seeded in 96‐well plate. At indicated time points, CCK‐8 reagents (Dojindo, Kumamoto, Japan) were added and incubated for another 1 h. Then, the absorbance values at 450 nm were detected using a microplate reader (Bio‐Rad, Hercules, CA, USA) to indicate cell proliferation. EdU incorporation experiments were conducted using the EdU Kit (Roche) following the provided protocol. The percentage of EdU‐positive cells was counted by a fluorescence microscope based on at least five randomly fields.

### 2.14 Cell cycle and apoptosis assays

Cell cycle of indicated HCC cells was detected by the Cell Cycle and Apoptosis Analysis Kit (Beyotime) following the provided protocol. After propidium iodide (PI) staining, cell cycle was detected using FACSCalibur (BD Biosciences, San Jose, CA, USA) based on red fluorescence at 488 nm. Cell apoptosis of indicated HCC cells was detected by the FITC‐Annexin V/PI Apoptosis Detection Kit (BD Biosciences) following the provided protocol on FACSCalibur.

### 2.15 Cell invasion assay

Cell invasion of indicated HCC cells was detected by the Cell Invasion Assay Kit (Cat. No. ECM550, Millipore) following the provided protocol. The number of invaded cells was counted by a microscope based on at least five randomly fields.

### 2.16 Animal studies

Male six‐week‐old athymic BALB/c nude mice were purchased from SLAC Laboratory Animal Co. Ltd (Shanghai, China). Animal experiments were conducted in accordance with the Animal Care and Use Committee guidelines of Henan University. Indicated HCC cells were subcutaneously injected into the flanks of nude mice at 2 × 10^6^ cells per site. Subcutaneous xenograft growth was recorded weekly by a caliper. The xenograft volume was calculated as a × b^2^ × 0.5 (a, long axes; b, short axes). At the 28th day after injection, the mice were sacrificed and the xenografts were resected, photographed, and weighed. For liver metastasis experiments, 2 × 10^6^ indicated HCC cells were intrasplenic injected into nude mice. The mice were bred for another 5 weeks. Then, the mice were sacrificed and the livers were resected. Liver metastasis was evaluated using hematoxylin and eosin (HE) staining. At least three random liver metastasis lesions from each group were subjected to RNA extraction. F4/80^+^ CD11b^+^ macrophages were sorted from liver metastasis lesions by magnetic‐activated cell sorting with anti‐F4/80 MicroBeads (Miltenyi, Auburn, CA, USA) and CD11b MicroBeads (Miltenyi) following the provided protocols.

### 2.17 Immunohistochemistry (IHC) and immunofluorescence (IF)

Paraffin‐embedded sections from subcutaneous xenografts were used to conduct immunohistochemistry (IHC) staining as previously described (Bao *et al.*, 2019) with primary antibodies against Ki67 (Abcam), cleaved caspase‐3 (Abcam), or WNT3A (Abcam). Paraffin‐embedded sections from human HCC tissues were used to conduct IHC staining with primary antibody against CD163 (Abcam). Paraffin‐embedded sections from liver metastasis lesions were used to conduct immunofluorescence (IF) staining as previously described (Yuan *et al.*, 2014) with primary antibodies against CD163 (Abcam).

### 2.18 Human HCC tissues

Eighty‐six pairs of HCC tissues and matched noncancerous liver tissues were obtained with written informed consent from HCC patients who received radical surgery at Henan Provincial People's Hospital (Zhengzhou, China). The clinical characteristics of these 86 cases are presented in Table 1. The study methodologies conformed to the standards set by the Declaration of Helsinki. This study was approved by the institutional review board of Henan Provincial People's Hospital.

**Table 1:** Clinical characteristics of 86 HCC patients according to LINC00662 expression levels. *P*‐value was acquired by Pearson’s chi‐square tests.

Patient characteristics	LINC00662		Chi‐square	*P*‐value
	Low	High		
Gender				
Male	38	39	0.124	0.725
Female	5	4		
Age				
≥ 50	23	19	0.754	0.388
< 50	20	24		
HB antigen				
Positive	36	38	0.387	0.534
Negative	7	5		
AFP (ng·mL^−1^)				
> 20	24	30	1.792	0.181
≤ 20	19	13		
Differentiation				
I‐II	10	3	4.440	**0.035**
III‐IV	33	40		
Tumor size (cm)				
> 5	18	29	5.677	**0.017**
≤ 5	25	14		
Microvascular invasion				
Present	17	28	5.640	**0.018**
Absent	26	15		
Tumor number				
Single	36	35	0.081	0.776
Multiple	7	8		

Bold values indicate *P* < 0.05.

### 2.19 Statistical analysis

graphpad prism 6.0 software was used to conduct statistical analyses. One‐way analysis of variance (ANOVA) followed by Dunnett's multiple comparisons test, Kruskal–Wallis test followed by Dunn's multiple comparisons test, Wilcoxon matched‐pairs signed rank test, log‐rank test, Pearson correlation analysis, Mann–Whitney test, and Pearson’s chi‐square test were conducted as indicated in the figure and table legends. *P* < 0.05 was considered to be significant.

## 3 Results

### 3.1 LINC00662 physically binds miR‐15a, miR‐16, and miR‐107

**Figure 1:**
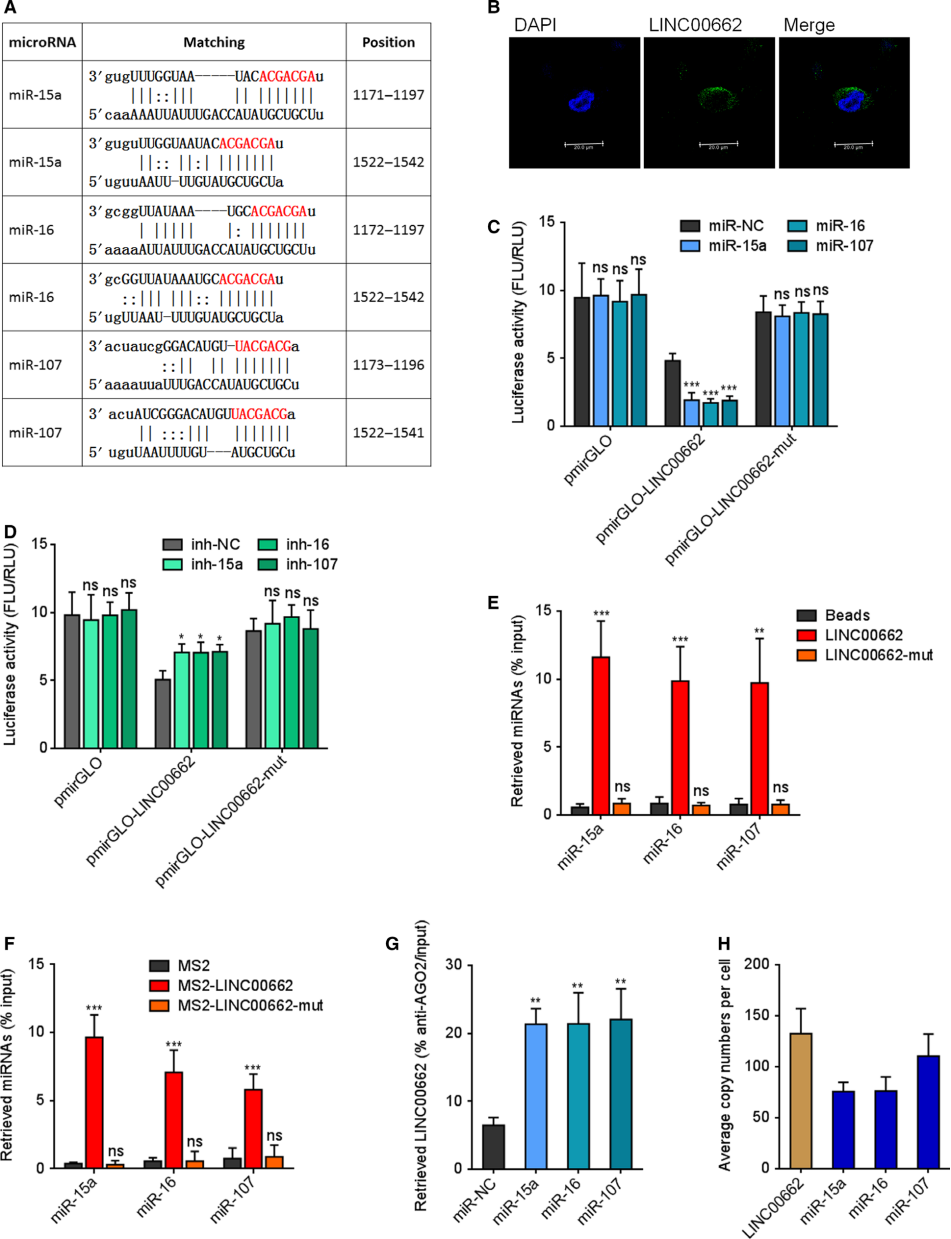
LINC00662 physically binds miR‐15a, miR‐16, and miR‐107. (A) The prediction for miR‐15a, miR‐16, and miR‐107 binding sites on LINC00662 transcript. The red nucleotides are the seed sequences of miRNAs. (B) Confocal RNA FISH images showed cytoplasmic localization of LINC00662. Scale bars, 20 µm. (C) Luciferase activity in HCCLM3 cells cotransfected with miR‐15a, miR‐16, or miR‐107 mimics and luciferase reporters containing nothing, wild‐type LINC00662, or miR‐15a/16/107 binding sites mutated LINC00662. Result is presented as the ratio of firefly luciferase activity to renilla luciferase activity. (D) Luciferase activity in HCCLM3 cells cotransfected with miR‐15a, miR‐16, or miR‐107 inhibitors and luciferase reporters containing nothing, wild‐type LINC00662, or mutated LINC00662. Result is presented as the ratio of firefly luciferase activity to renilla luciferase activity. (E) HCCLM3 cell lysates were incubated with biotin‐labeled wild‐type or mutated LINC00662; after pull‐down, RNAs were extracted and measured by qRT/PCR. (F) After transient coexpression of MS2‐GFP fusion protein and MS2‐LINC00662 fusion transcript, anti‐GFP RIP was undertaken, followed by qRT/PCR to detect miRNAs endogenously associated with LINC00662. (G) After transient transfection of miR‐15a, miR‐16, or miR‐107 mimics into HCCLM3 cells, anti‐AGO2 RIP was performed, followed by qRT/PCR to detect LINC00662 associated with AGO2. (H) The exact expression levels of LINC00662, miR‐15a, miR‐16, and miR‐107 in HCCLM3 cells were detected with quantitative RT/PCR. Results are shown as mean ± standard error based on three independent experiments. *P < 0.05, **P < 0.01, ***P < 0.001; ns, not significant, by one‐way ANOVA followed by Dunnett's multiple comparisons test.

miR‐15a, miR‐16, and miR‐107 are classical miRNAs which target WNT3A (Bonci *et al.*, 2008; Li *et al.*, 2018). Therefore, we searched the lncRNAs which physically binds miR‐15a, miR‐16, and miR‐107 using ENCOPI (http://starbase.sysu.edu.cn/). ENCOPI predicted the potential miRNA–lncRNA interactions using Ago CLIP‐seq data and miRanda program. Among the predicted lncRNAs, we noted LINC00662 which has two predicted interaction regions with miR‐15a/16/107 (Fig. 1A). Furthermore, the TCGA data reveal that high expression of LINC00662 is associated with poor overall survival of liver hepatocellular carcinoma (LIHC) patients (Fig. S1A), and LINC00662 is highly expressed in LIHC tissues compared with normal liver tissues (Fig. S1B). GEO data also reveal that LINC00662 (probe number 1558256_at) is increased in HCC tissues compared with liver tissues (Fig. S1C). Northern blot experiments confirmed the expected size of LINC00662 and that the NR_027301 (NCBI Reference Sequence Number) is the main transcript of LINC00662 (Fig. S1D). The expression of LINC00662 is markedly increased in HCC cells HCCLM3, MHCC97H, Huh7, and SK‐HEP‐1, compared with normal liver cell QSG‐7701 (Fig. S1E). RNA FISH assay and cytoplasmic and nuclear RNA isolation assay reveal that LINC00662 is mainly located in the cytoplasm (Fig. 1B and Fig. S1F).

LINC00662 sequences containing the predicted miR‐15a/16/107 binding sites or the same LINC00662 sequences with miR‐15a/16/107 binding sites mutated were cloned into the 3'UTR of firefly luciferase encoding gene in luciferase reporters. Dual‐luciferase reporter experiments presented that overexpression of miR‐15a, miR‐16, or miR‐107 markedly reduced the luciferase activities of the constructed reporter vector, which was abolished by the mutation of miR‐15a/16/107 binding sites (Fig. 1C). Inhibition of miR‐15a, miR‐16, or miR‐107 increased the luciferase activities of the constructed reporter vector, which was also abolished by the mutation of miR‐15a/16/107 binding sites (Fig. 1D). RNA–RNA pull‐down experiments were performed using *in vitro*‐transcribed LINC00662 to enrich interacted miRNAs. As presented in Fig. 1E, miR‐15a, miR‐16, and miR‐107 were significantly enriched in LINC00662 group. Moreover, miR‐15a/16/107 binding sites mutated LINC00662 was also *in vitro*‐transcribed to perform RNA–RNA pull‐down experiments. The data presented that the mutation of miR‐15a/16/107 binding sites abolished the enrichment of miR‐15a/16/107 (Fig. 1E). To further confirm the interaction between LINC00662 and miR‐15a, miR‐16, miR‐107, MS2 RIP experiments were performed to enrich the RNAs bound to LINC00662. As presented in Fig. 1F, compared with empty vector (MS2) group, miR‐15a, miR‐16, and miR‐107 were significantly enriched in LINC00662 group. Consistently, the mutation of miR‐15a/16/107 binding sites abolished the enrichment of miR‐15a/16/107 (Fig. 1F). miRNAs are well known to bind AGO2 to form RNA‐induced silencing complex (RISC) and then co‐bind targets (Chandradoss *et al.*, 2015). Thus, anti‐AGO2 RIP experiments were undertaken after transient overexpression of miR‐15a, miR‐16, or miR‐107. As presented in Fig. 1G, overexpression of miR‐15a, miR‐16, and miR‐107 markedly increased the binding between LINC00662 and AGO2. To act as a ceRNA, the expression levels of lncRNAs and miRNAs should be comparable in cells. The exact expression levels of LINC00662, miR‐15a, miR‐16, and miR‐107 were quantified in HCCLM3 cells. As presented in Fig. 1H, the expression levels of LINC00662, miR‐15a, miR‐16, and miR‐107 were approximately 100 copies per cell. All these results suggested that LINC00662 physically binds miR‐15a, miR‐16, and miR‐107.

### 3.2 LINC00662 upregulates WNT3A level and activates Wnt/β‐catenin signaling in an autocrine manner

**Figure 2:**
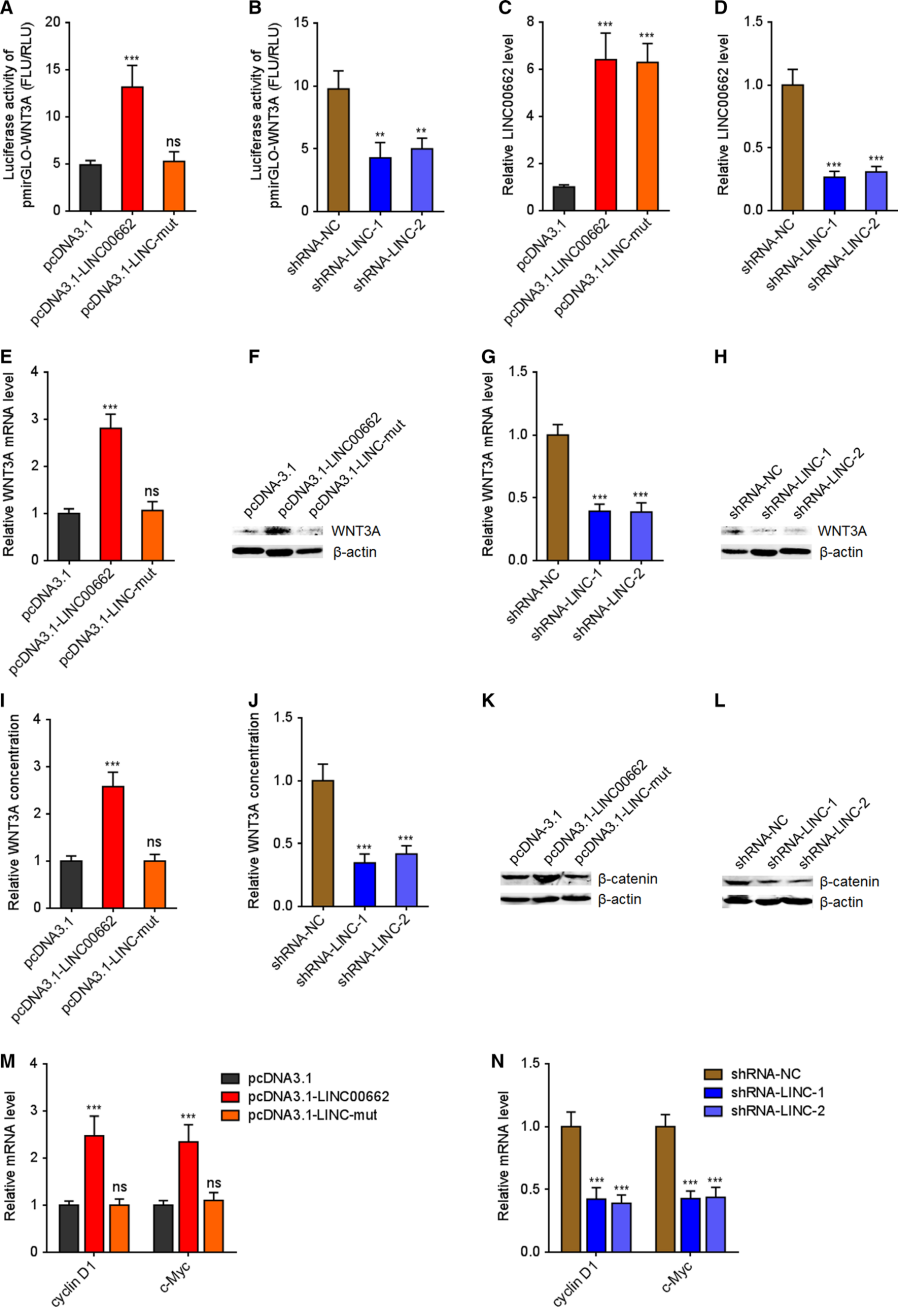
LINC00662 upregulates WNT3A level and activates Wnt/β‐catenin signaling in an autocrine manner. (A) Luciferase activity in HCCLM3 cells cotransfected with wild‐type LINC00662, or miR‐15a/16/107 binding sites mutated LINC00662 overexpression plasmids and luciferase reporter containing 3'UTR of WNT3A. Result is presented as the ratio of firefly luciferase activity to renilla luciferase activity. (B) Luciferase activity in SK‐HEP‐1 cells cotransfected with LINC00662‐specific shRNAs and luciferase reporter containing 3'UTR of WNT3A. Result is presented as the ratio of firefly luciferase activity to renilla luciferase activity. (C) LINC00662 expression in wild‐type or mutated LINC00662 stably overexpressed and control HCCLM3 cells was measured by qRT/PCR. (D) LINC00662 expression in LINC00662 stably silenced and control SK‐HEP‐1 cells was measured by qRT/PCR. (E) WNT3A mRNA levels in wild‐type or mutated LINC00662 stably overexpressed and control HCCLM3 cells were measured by qRT/PCR. (F) WNT3A protein levels in wild‐type or mutated LINC00662 stably overexpressed and control HCCLM3 cells were measured by western blot. (G) WNT3A mRNA levels in LINC00662 stably silenced and control SK‐HEP‐1 cells were measured by qRT/PCR. (H) WNT3A protein levels in LINC00662 stably silenced and control SK‐HEP‐1 cells were measured by western blot. (I) Concentrations of WNT3A in the culture supernatants of wild‐type or mutated LINC00662 stably overexpressed and control HCCLM3 cells were measured by ELISA. (J) Concentrations of WNT3A in the culture supernatants of LINC00662 stably silenced and control SK‐HEP‐1 cells were measured by ELISA. (K) β‐Catenin protein levels in wild‐type or mutated LINC00662 stably overexpressed and control HCCLM3 cells were measured by western blot. (L) β‐Catenin protein levels in LINC00662 stably silenced and control SK‐HEP‐1 cells were measured by western blot. (M) Wnt/β‐catenin signaling target genes cyclin D1 and c‐Myc mRNA levels in wild‐type or mutated LINC00662 stably overexpressed and control HCCLM3 cells were measured by qRT/PCR. (N) Cyclin D1 and c‐Myc mRNA levels in LINC00662 stably silenced and control SK‐HEP‐1 cells were measured by qRT/PCR. Results are shown as mean ± standard error based on three independent experiments. **P < 0.01, ***P < 0.001; ns, not significant, by one‐way ANOVA followed by Dunnett's multiple comparisons test.

Due to LINC00662 shares miR‐15a, miR‐16, and miR‐107 with WNT3A, we next investigated whether LINC00662 regulates WNT3A in a ceRNA manner. 3'UTR of WNT3A containing the reported miR‐15a/16/107 binding sites was cloned into the 3'UTR of firefly luciferase encoding gene in luciferase reporters. Dual‐luciferase reporter experiments presented that simultaneous overexpression of LINC00662 significantly increased the luciferase activities of the constructed reporter vector (Fig. 2A). The mutation of miR‐15a/16/107 binding sites on LINC00662 abolished the effects of LINC00662 overexpression (Fig. 2A). Conversely, LINC00662 silencing reduced the luciferase activities of the constructed reporter vector (Fig. 2B). Then, the mRNA and protein expression levels of WNT3A in LINC00662 stably overexpressed and silenced HCC cells were detected. Wild‐type LINC00662 or miR‐15a/16/107 binding sites mutated LINC00662 overexpression vectors were transfected into HCCLM3 cells to construct wild‐type or mutated LINC00662 stably overexpressed cells with similar overexpression efficiencies (Fig. 2C). Two independent LINC00662 specific shRNAs were transfected into SK‐HEP‐1 cells to construct LINC00662 stably silenced cells (Fig. 2D). Overexpression of LINC00662 markedly upregulated WNT3A mRNA and protein levels, which was abolished by the mutation of miR‐15a/16/107 binding sites (Fig. 2E,F). LINC00662 silencing markedly downregulated WNT3A mRNA and protein levels (Fig. 2G,H).

WNT3A is a critical Wnt ligand which could be secreted from tumors cell and activate Wnt/β‐catenin signaling (Yang *et al.*, 2018). Therefore, we explored whether LINC00662 modulates the secretion of WNT3A. WNT3A levels in the cell supernatants of LINC00662 stably overexpressed and silenced HCC cells were detected by ELISA. As presented in Fig. 2I, overexpression of LINC00662 upregulated WNT3A concentration which was abolished by the mutation of miR‐15a/16/107 binding sites. LINC00662 silencing downregulated WNT3A concentration (Fig. 2J). To investigate whether LINC00662 could activate Wnt/β‐catenin signaling in an autocrine manner via promoting WNT3A secretion, β‐catenin protein level was detected in LINC00662 stably overexpressed and silenced HCC cells. The data presented that overexpression of LINC00662 upregulated β‐catenin protein level, which was abolished by the mutation of miR‐15a/16/107 binding sites (Fig. 2K). Conversely, LINC00662 silencing downregulated β‐catenin protein level (Fig. 2L). Wnt/β‐catenin signaling target genes, such as cyclin D1 and c‐Myc, were also upregulated in LINC00662‐overexpressed HCC cells, which were abolished by the mutation of miR‐15a/16/107 binding sites (Fig. 2M). Consistently, cyclin D1 and c‐Myc were downregulated in LINC00662‐silenced HCC cells (Fig. 2N). All these results suggested that LINC00662 activates Wnt/β‐catenin signaling in an autocrine manner via competitively binding miR‐15a/16/107 and upregulating WNT3A.

### 3.3 LINC00662 promotes HCC cell proliferation, cell cycle, and invasion and represses cell apoptosis

**Figure 3:**
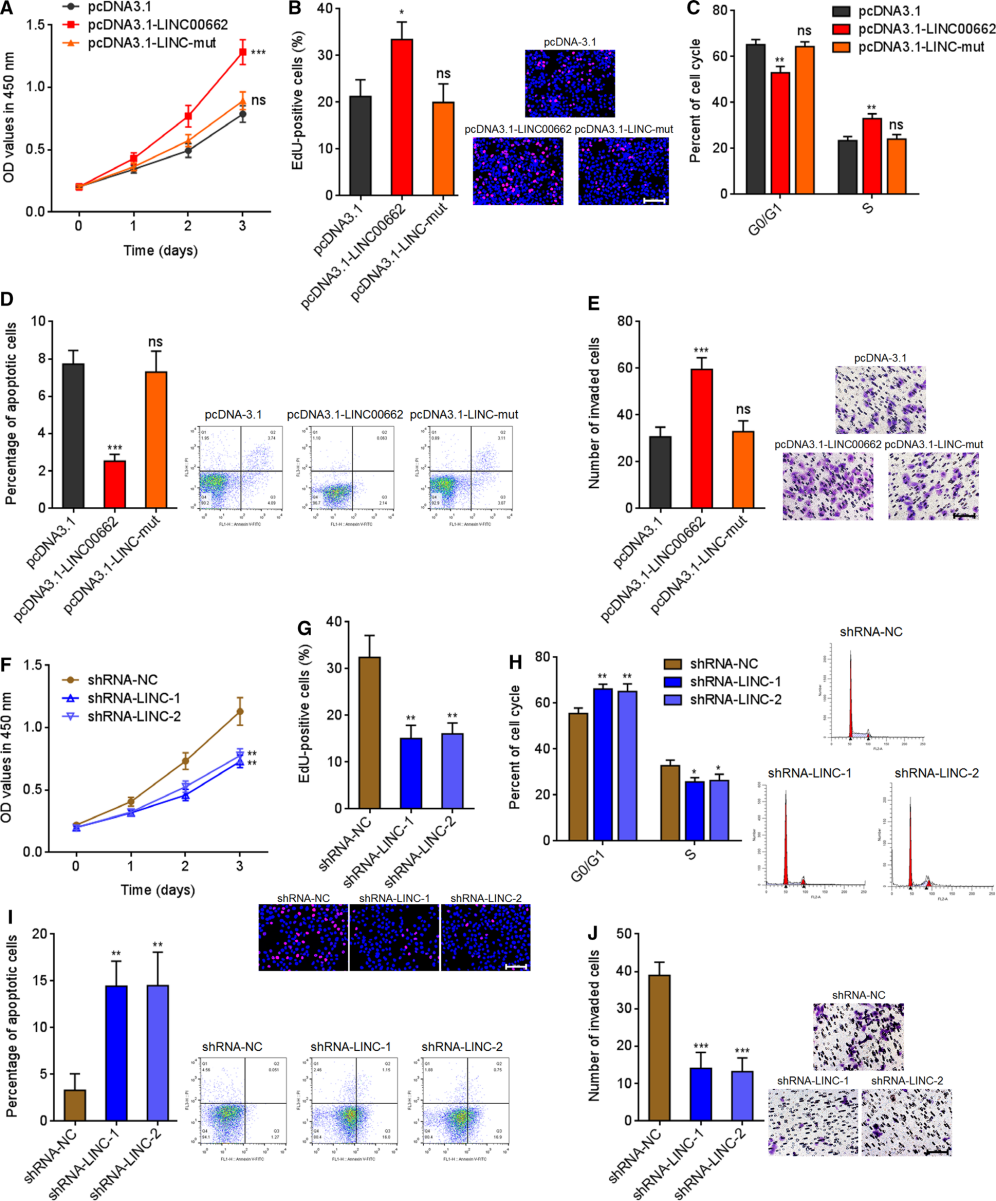
LINC00662 promotes HCC cell proliferation, cell cycle, and invasion and represses cell apoptosis. (A) Cell proliferation of wild‐type or mutated LINC00662 stably overexpressed and control HCCLM3 cells was detected by CCK‐8 assays. OD values in 450 nm were collected to indicate cell proliferation. (B) Cell proliferation of wild‐type or mutated LINC00662 stably overexpressed and control HCCLM3 cells was detected by EdU incorporation experiments. The red colors indicate EdU‐positive nuclei and proliferative cells. Scale bars = 100 µm. (C) The cell numbers of wild‐type or mutated LINC00662 stably overexpressed and control HCCLM3 cells in G0/G1 and S phages were determined by PI staining and FACS. (D) Apoptosis of wild‐type or mutated LINC00662 stably overexpressed and control HCCLM3 cells was measured by Annexin V‐PI staining and flow cytometry. (E) Invasion of wild‐type or mutated LINC00662 stably overexpressed and control HCCLM3 cells was measured by transwell invasion assays. Scale bars = 100 µm. (F) Cell proliferation of LINC00662 stably silenced and control SK‐HEP‐1 cells was detected by CCK‐8 assays. OD values in 450 nm were collected to indicate cell proliferation. (G) Cell proliferation of LINC00662 stably silenced and control SK‐HEP‐1 cells were detected by EdU incorporation experiments. The red colors indicate EdU‐positive nuclei and proliferative cells. Scale bars = 100 µm. (H) The cell numbers of LINC00662 stably silenced and control SK‐HEP‐1 cells in G0/G1 and S phages were determined by PI staining and FACS. (I) Apoptosis of LINC00662 stably silenced and control SK‐HEP‐1 cells was measured by Annexin V‐PI staining and flow cytometry. (J) Invasion of LINC00662 stably silenced and control SK‐HEP‐1 cells was measured by transwell invasion assays. Scale bars = 100 µm. Results are shown as mean ± standard error based on three independent experiments. *P < 0.05, **P < 0.01, ***P < 0.001; ns, not significant, by one‐way ANOVA followed by Dunnett's multiple comparisons test.

Because Wnt/β‐catenin signaling has critical roles in HCC (Shang *et al.*, 2019), we next explored the functions of LINC00662 in HCC. CCK‐8 and EdU incorporation experiments both presented that overexpression of LINC00662 accelerated HCCLM3 cell proliferation, which was abolished by the mutation of miR‐15a/16/107 binding sites (Fig. 3A,B). Cell cycle analysis revealed that overexpression of LINC00662 promoted G1/S cell cycle progression, which was abolished by the mutation of miR‐15a/16/107 binding sites (Fig. 3C). Flow cytometry presented that overexpression of LINC00662 repressed HCCLM3 cell apoptosis, which was abolished by the mutation of miR‐15a/16/107 binding sites (Fig. 3D). Transwell invasion experiments presented that overexpression of LINC00662 accelerated HCCLM3 cell invasion, which was abolished by the mutation of miR‐15a/16/107 binding sites (Fig. 3E). Wild‐type LINC00662 or miR‐15a/16/107 binding sites mutated LINC00662 were stably overexpressed in another HCC cell line MHCC97H (Fig. S2A). Consistently, overexpression of LINC00662 accelerated MHCC97H cell proliferation, G1/S cell cycle progression, and cell invasion, and repressed MHCC97H cell apoptosis, which were abolished by the mutation of miR‐15a/16/107 binding sites (Fig. S2B–F). The functions of LINC00662 silencing were further elucidated in HCC cells. LINC00662 silencing repressed SK‐HEP‐1 cell proliferation (Fig. 3F,G). LINC00662 silencing induced G0/G1 cell cycle arrest (Fig. 3H). LINC00662 silencing promoted SK‐HEP‐1 cell apoptosis (Fig. 3I). LINC00662 silencing repressed SK‐HEP‐1 cell invasion (Fig. 3J). LINC000662 was further silenced in another HCC cell line Huh7 (Fig. S2G). Consistently, LINC00662 silencing repressed Huh7 cell proliferation, induced G0/G1 cell cycle arrest and cell apoptosis, and repressed Huh7 cell invasion (Fig. S2H–L). These results suggested that LINC00662 has oncogenic roles in HCC cell proliferation, cell cycle, apoptosis, and invasion via competitively binding miR‐15a/16/107.

### 3.4 LINC00662 promotes hepatic tumor growth via activating Wnt/β‐catenin signaling

**Figure 4:**
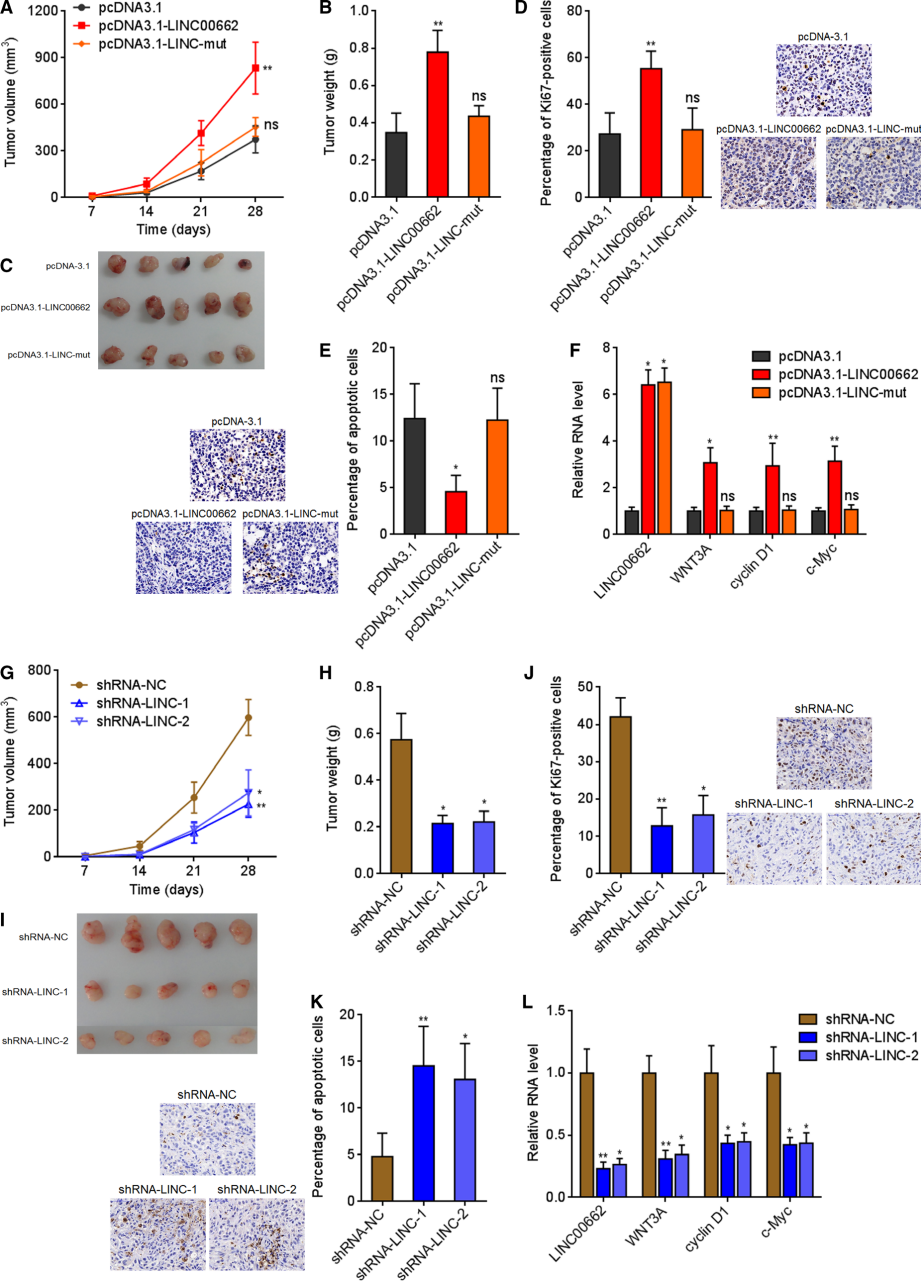
LINC00662 promotes hepatic tumor growth via activating Wnt/β‐catenin signaling. (A) Wild‐type or mutated LINC00662 stably overexpressed and control HCCLM3 cells were subcutaneously inoculated into nude mice. Tumor volumes were measured every 7 days. (B, C) The mice were sacrificed, and subcutaneous tumors were excised and weighed at the 28th day after inoculation. (D) Ki67 IHC staining of tumors derived from (C). (E) Cleaved caspase‐3 IHC staining of tumors derived from (C). (F) The expression of LINC00662, WNT3A, cyclin D1, and c‐Myc in the tumors derived from (C) was measured by qRT/PCR. (G) LINC00662 stably silenced and control SK‐HEP‐1 cells were subcutaneously inoculated into nude mice. Tumor volumes were measured every 7 days. (H, I) The mice were sacrificed, and subcutaneous tumors were excised and weighed at the 28th day after inoculation. (J) Ki67 IHC staining of tumors derived from (I). (K) Cleaved caspase‐3 IHC staining of tumors derived from (I). (L) The expression of LINC00662, WNT3A, cyclin D1, and c‐Myc in the tumors derived from (I) was measured by qRT/PCR. Results are shown as mean ± standard error based on *n* = 5 mice in each group. *P < 0.05, **P < 0.01, ns, not significant, by Kruskal–Wallis test followed by Dunn's multiple comparisons test.

The functions of LINC00662 were further elucidated in hepatic tumor growth *in vivo*. Wild‐type or miR‐15a/16/107 binding sites mutated LINC00662 stably overexpressed HCCLM3 cells were subcutaneously inoculated into nude mice. As presented in Fig. 4A–C, overexpression of LINC00662 markedly accelerated xenograft growth *in vivo*. Proliferation marker Ki67 IHC staining showed that the xenograft derived from wild‐type LINC00662, but not mutated LINC00662‐overexpressed cells had more proliferative cells (Fig. 4D). Apoptosis marker cleaved caspase‐3 IHC staining showed that the xenograft derived from wild‐type LINC00662, but not mutated LINC00662‐overexpressed cells had less apoptotic cells (Fig. 4E). The overexpression efficiencies of LINC00662 were confirmed in the xenograft (Fig. 4F). LINC00662 also upregulated WNT3A and activated Wnt/β‐catenin signaling in the xenograft in a miR‐15a/16/107 dependent manner, as documented by the upregulation of cyclin D1 and c‐Myc (Fig. 4F and Fig. S3A). To further confirm the roles of LINC00662 in hepatic tumor growth *in vivo*, LINC00662 stably silenced SK‐HEP‐1 cells were subcutaneously inoculated into nude mice. As presented in Fig. 4G–I, LINC00662 silencing markedly repressed xenograft growth *in vivo*. Ki67 IHC staining showed that the xenograft derived from LINC00662‐silenced cells had less proliferative cells (Fig. 4J). Cleaved caspase‐3 IHC staining showed that the xenograft derived from LINC00662‐silenced cells had more apoptotic cells (Fig. 4K). The silencing efficiencies of LINC00662 were confirmed in the xenograft (Fig. 4L). LINC00662 silencing also downregulated WNT3A and repressed Wnt/β‐catenin signaling in the xenograft, as documented by the downregulation of cyclin D1 and c‐Myc (Fig. 4L and Fig. S3B). These results suggested that LINC00662 upregulates WNT3A, activates Wnt/β‐catenin signaling, and further promotes hepatic tumor growth *in vivo* in a miR‐15a/16/107‐dependent manner.

### 3.5 LINC00662 induces M2 macrophage polarization via activating Wnt/β‐catenin signaling in a paracrine manner

**Figure 5:**
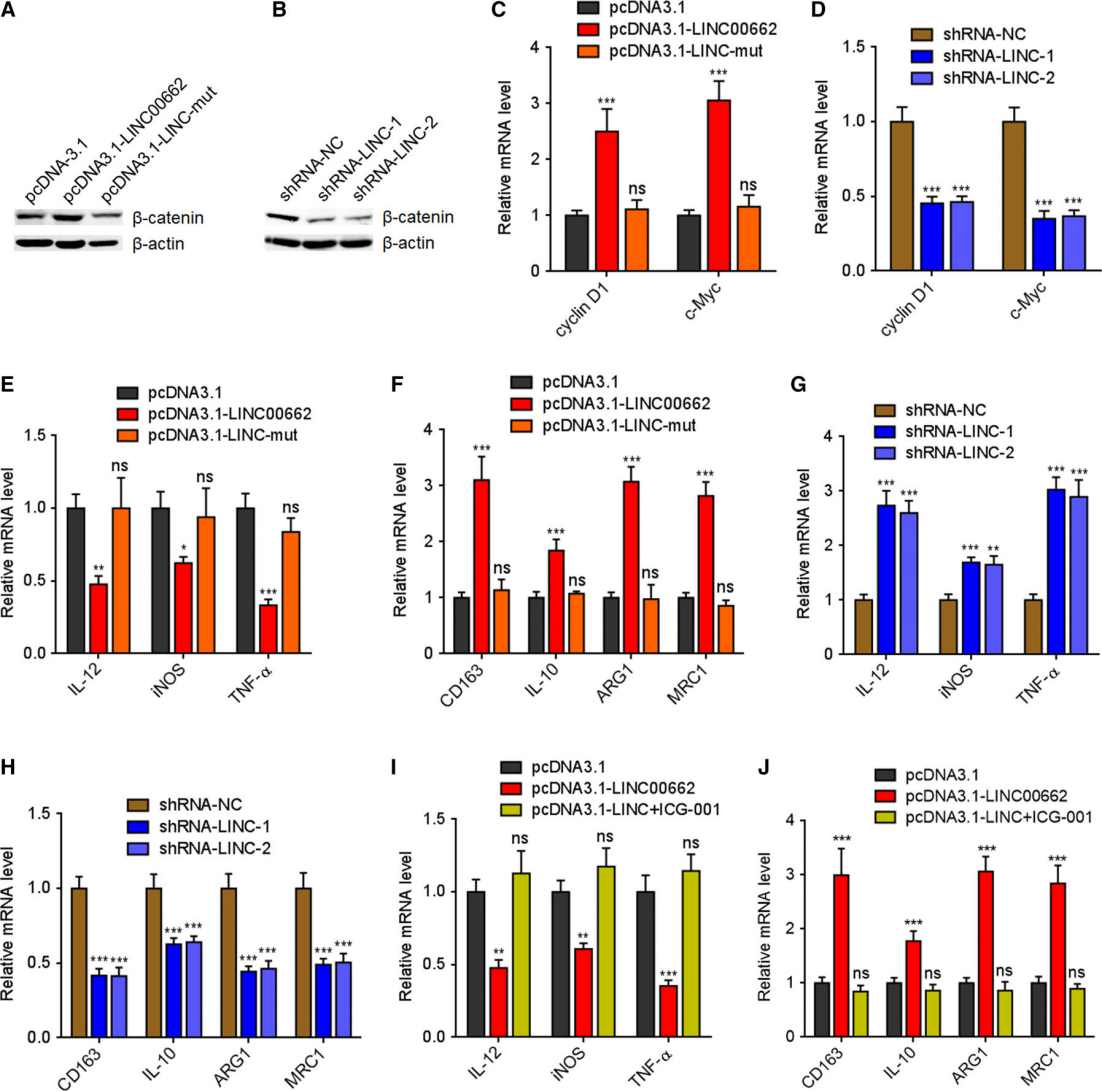
LINC00662 induces M2 macrophages polarization via activating Wnt/β‐catenin signaling in a paracrine manner. (A) β‐Catenin protein levels in THP‐1 cells treated with conditional medium from wild‐type or mutated LINC00662 stably overexpressed or control HCCLM3 cells were measured by western blot. (B) β‐Catenin protein levels in THP‐1 cells treated with conditional medium from LINC00662 stably silenced or control SK‐HEP‐1 cells were measured by western blot. (C) Wnt/β‐catenin signaling target genes cyclin D1 and c‐Myc mRNA levels in THP‐1 cells treated with conditional medium from wild‐type or mutated LINC00662 stably overexpressed or control HCCLM3 cells were measured by qRT/PCR. (D) cyclin D1 and c‐Myc mRNA levels in THP‐1 cells treated with conditional medium from LINC00662 stably silenced or control SK‐HEP‐1 cells were measured by qRT/PCR. (E) M1 macrophage surface markers IL‐12, iNOS, and TNF‐α mRNA levels in THP‐1 cells treated with conditional medium from wild‐type or mutated LINC00662 stably overexpressed or control HCCLM3 cells were measured by qRT/PCR. (F) M2 macrophage surface markers CD163, IL‐10, ARG1, and MRC1 mRNA levels in THP‐1 cells treated with conditional medium from wild‐type or mutated LINC00662 stably overexpressed or control HCCLM3 cells were measured by qRT/PCR. (G) IL‐12, iNOS, and TNF‐α mRNA levels in THP‐1 cells treated with conditional medium from LINC00662 stably silenced or control SK‐HEP‐1 cells were measured by qRT/PCR. (H) CD163, IL‐10, ARG1, and MRC1 mRNA levels in THP‐1 cells treated with conditional medium from LINC00662 stably silenced or control SK‐HEP‐1 cells were measured by qRT/PCR. (I) IL‐12, iNOS, and TNF‐α mRNA levels in THP‐1 cells treated with conditional medium from LINC00662 stably overexpressed HCCLM3 cells supplemented with 25 μm ICG‐001 were measured by qRT/PCR. (J) CD163, IL‐10, ARG1, and MRC1 mRNA levels in THP‐1 cells treated with conditional medium from LINC00662 stably overexpressed HCCLM3 cells supplemented with 25 μm ICG‐001 were measured by qRT/PCR. Results are shown as mean ± standard error based on three independent experiments. *P < 0.05, **P < 0.01, ***P < 0.001, ns, not significant, by one‐way ANOVA followed by Dunnett's multiple comparisons test.

Except to the activation of Wnt/β‐catenin signaling in tumor cells, WNT3A has also been reported to activate Wnt/β‐catenin signaling in macrophages and induce M2 macrophages polarization (Feng *et al.*, 2018; Yang *et al.*, 2018). Our data have demonstrated that LINC00662 upregulated WNT3A expression and increased WNT3A secretion from HCC cells. Therefore, we explored whether the increased expression of LINC00662 in HCC cells modulate M2 macrophages. THP‐1 differentiated macrophages were treated with conditional medium from wild‐type or miR‐15a/16/107 binding sites mutated LINC00662 stably overexpressed HCCLM3 cells, or conditional medium (CM) from LINC00662 stably silenced SK‐HEP‐1 cells. Our data presented that β‐catenin protein level was increased in THP‐1 macrophages treated with CM from wild‐type, but not mutated LINC00662‐overexpressed cells (Fig. 5A). β‐Catenin protein level was decreased in THP‐1 macrophages treated with CM from LINC00662‐silenced cells (Fig. 5B). Wnt/β‐catenin signaling target genes, such as cyclin D1 and c‐Myc, were upregulated in THP‐1 macrophages treated with CM from wild‐type, but not mutated LINC00662‐overexpressed cells (Fig. 5C). Conversely, cyclin D1 and c‐Myc were downregulated in THP‐1 macrophages treated with CM from LINC00662‐silenced cells (Fig. 5D). Moreover, M1 macrophage surface markers such as IL‐12, iNOS, and TNF‐α were reduced in THP‐1 macrophages treated with CM from wild‐type, but not mutated LINC00662‐overexpressed cells (Fig. 5E). M2 macrophage surface markers such as CD163, IL‐10, ARG1, and MRC1 were increased in THP‐1 macrophages treated with CM from wild‐type, but not mutated LINC00662‐overexpressed cells (Fig. 5F). Conversely, IL‐12, iNOS, and TNF‐α were increased in THP‐1 macrophages treated with CM from LINC00662‐silenced cells (Fig. 5G). CD163, IL‐10, ARG1, and MRC1 were reduced in THP‐1 macrophages treated with CM from LINC00662‐silenced cells (Fig. 5H). Thus, these data demonstrated that LINC00662 overexpression in HCC cells activates Wnt/β‐catenin signaling in macrophages and induces M2 macrophage polarization. To investigate whether the roles of LINC00662 in M2 macrophage polarization were dependent on the activation of Wnt/β‐catenin signaling, THP‐1‐differentiated macrophages were treated with Wnt signaling inhibitor ICG‐001. ICG‐001 reversed the reduction of M1 macrophage surface markers IL‐12, iNOS, and TNF‐α in THP‐1 macrophages treated with CM from LINC00662‐overexpressed cells (Fig. 5I). ICG‐001 also abolished the increasing of M2 macrophage surface markers CD163, IL‐10, ARG1, and MRC1 in THP‐1 macrophages treated with CM from LINC00662‐overexpressed cells (Fig. 5J). Collectively, these data suggested that LINC00662 activates Wnt/β‐catenin signaling in macrophages and induces M2 macrophages polarization in a paracrine manner.

### 3.6 LINC00662 promotes HCC liver metastasis via activating Wnt/β‐catenin signaling and inducing M2 macrophage polarization

**Figure 6:**
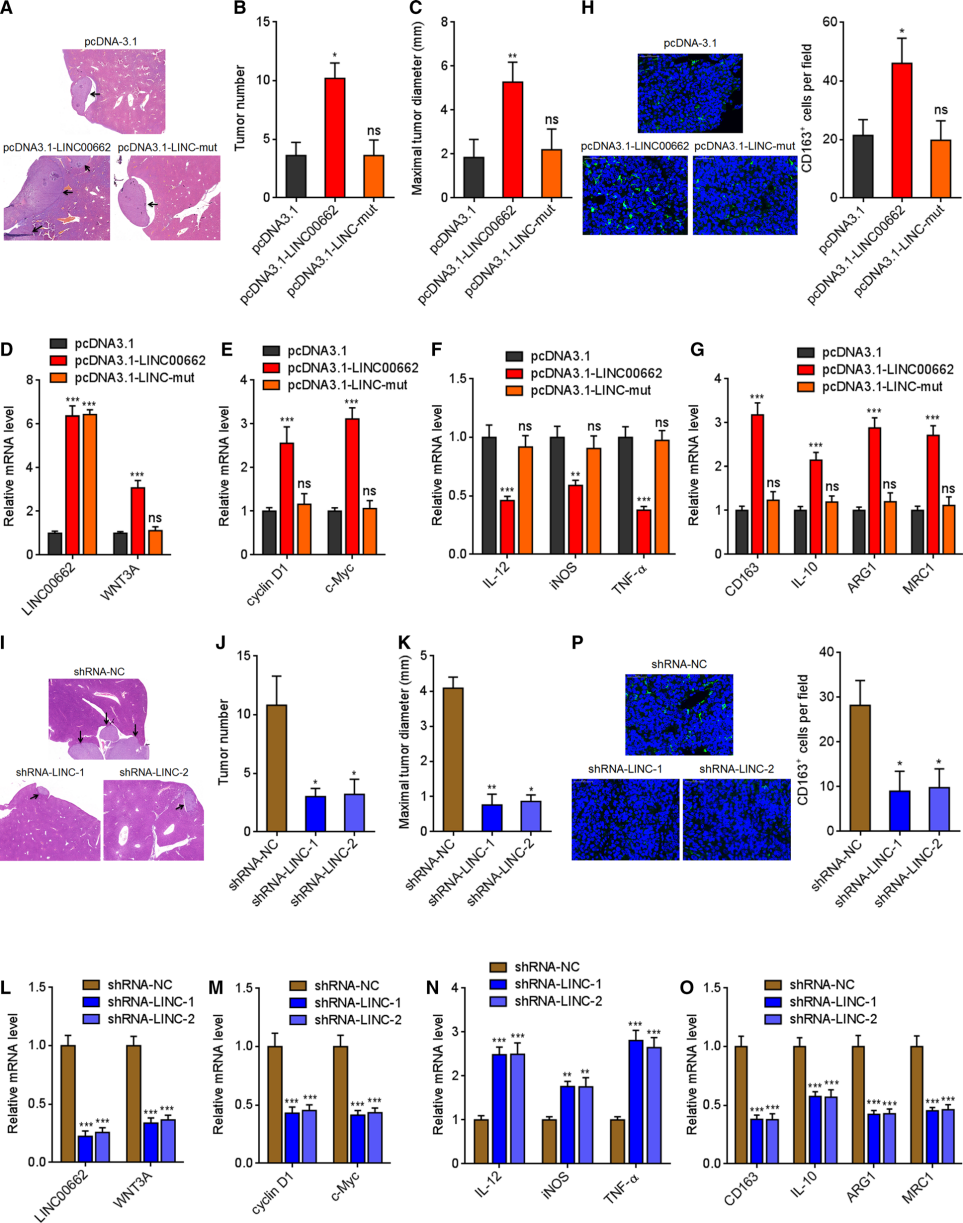
LINC00662 promotes HCC liver metastasis via activating Wnt/β‐catenin signaling and inducing M2 macrophage polarization. (A–C) Wild‐type or mutated LINC00662 stably overexpressed and control HCCLM3 cells were intrasplenic injected into nude mice. At the 35th day after injection, liver metastasis was measured by HE staining. (D) The expression of LINC00662 and WNT3A in the tumors derived from (A) was measured by qRT/PCR. (E–G) F4/80^+^ CD11b^+^ macrophages were isolated from the tumors derived from (A). The expression of Wnt/β‐catenin signaling target genes cyclin D1 and c‐Myc (E), M1 macrophage surface markers IL‐12, iNOS, and TNF‐α (F), and M2 macrophage surface markers CD163, IL‐10, ARG1, and MRC1 (G) was measured by qRT/PCR. (H) CD163 IF staining of tumors derived from (A). (I–K) LINC00662 stably silenced and control SK‐HEP‐1 cells were intrasplenic injected into nude mice. At the 35th day after injection, liver metastasis was measured by HE staining. (L) The expression of LINC00662 and WNT3A in the tumors derived from (I) was measured by qRT/PCR. (M–O) F4/80^+^ CD11b^+^ macrophages were isolated from the tumors derived from (I). The expression of Wnt/β‐catenin signaling target genes cyclin D1 and c‐Myc (M), M1 macrophage surface markers IL‐12, iNOS, and TNF‐α (N), and M2 macrophage surface markers CD163, IL‐10, ARG1, and MRC1 (O) was measured by qRT/PCR. (P) CD163 IF staining of tumors derived from (I). For A‐C, H‐K, and P, results are shown as mean ± standard error based on n = 5 mice in each group. *P < 0.05, **P < 0.01, ns, not significant, by Kruskal–Wallis test followed by Dunn's multiple comparisons test. For other panels, results are shown as mean ± standard error based on three random tumors in each group. **P < 0.01, ***P < 0.001, ns, not significant, by one‐way ANOVA followed by Dunnett's multiple comparisons test.

To investigate the effects of LINC00662 on macrophage *in vivo*, we employed intrasplenic injection‐liver metastasis model. Wild‐type or miR‐15a/16/107 binding sites mutated LINC00662 stably overexpressed HCCLM3 cells were injected into the spleen of nude mice. As presented in Fig. 6A–C, wild‐type LINC00662, but not mutated LINC00662‐overexpressed HCCLM3 cells formed more and larger liver metastatic foci. The overexpression efficiencies of LINC00662 were confirmed in the liver metastatic foci (Fig. 6D). Consistently, LINC00662 also upregulated WNT3A in the liver metastatic foci in a miR‐15a/16/107‐dependent manner (Fig. 6D). F4/80^+^ CD11b^+^ macrophages were isolated from liver metastatic foci, and the expression of Wnt/β‐catenin signaling target genes and M1 and M2 macrophage surface markers was detected. Wnt/β‐catenin signaling target genes, such as cyclin D1 and c‐Myc, were increased in macrophages isolated from liver metastatic foci formed by wild‐type LINC00662, but not mutated LINC00662‐overexpressed HCCLM3 cells (Fig. 6E). M1 macrophage surface markers such as IL‐12, iNOS, and TNF‐α were reduced in macrophages isolated from liver metastatic foci formed by wild‐type LINC00662, but not mutated LINC00662‐overexpressed HCCLM3 cells (Fig. 6F). M2 macrophage surface markers such as CD163, IL‐10, ARG1, and MRC1 were increased in macrophages isolated from liver metastatic foci formed by wild‐type LINC00662, but not mutated LINC00662‐overexpressed HCCLM3 cells (Fig. 6G). In addition, IF experiments presented that liver metastatic foci formed by wild‐type LINC00662, but not mutated LINC00662‐overexpressed HCCLM3 cells had more CD163‐positive M2 macrophages (Fig. 6H). To further confirm the roles of LINC00662 in HCC metastasis and macrophage polarization *in vivo*, LINC00662 stably silenced SK‐HEP‐1 cells were injected into the spleen of nude mice. As presented in Fig. 6I‐K, LINC00662‐silenced SK‐HEP‐1 cells formed less and smaller liver metastatic foci. The silencing efficiencies of LINC00662 were confirmed in the liver metastatic foci (Fig. 6L). Consistently, LINC00662 silencing downregulated WNT3A in the liver metastatic foci (Fig. 6L). F4/80^+^ CD11b^+^ macrophages were isolated from liver metastatic foci, and the expression of Wnt/β‐catenin signaling target genes and M1 and M2 macrophage surface markers was detected. Wnt/β‐catenin signaling target genes, such as cyclin D1 and c‐Myc, were reduced in macrophages isolated from liver metastatic foci formed by LINC00662‐silenced SK‐HEP‐1 cells (Fig. 6M). M1 macrophage surface markers such as IL‐12, iNOS, and TNF‐α were increased in macrophages isolated from liver metastatic foci formed by LINC00662‐silenced SK‐HEP‐1 cells (Fig. 6N). M2 macrophage surface markers such as CD163, IL‐10, ARG1, and MRC1 were reduced in macrophages isolated from liver metastatic foci formed by LINC00662‐silenced SK‐HEP‐1 cells (Fig. 6O). Furthermore, IF assays presented that liver metastatic foci formed by LINC00662 silenced SK‐HEP‐1 cells had less CD163‐positive M2 macrophages (Fig. 6P). These results suggested that LINC00662 activates Wnt/β‐catenin signaling in macrophages, induces M2 macrophage polarization, and further promotes HCC metastasis *in vivo* in a miR‐15a/16/107‐dependent manner.

### 3.7 LINC00662 is increased in HCC tissues and associated with WNT3A expression, M2 macrophage polarization, and poor outcome of HCC patients

**Figure 7:**
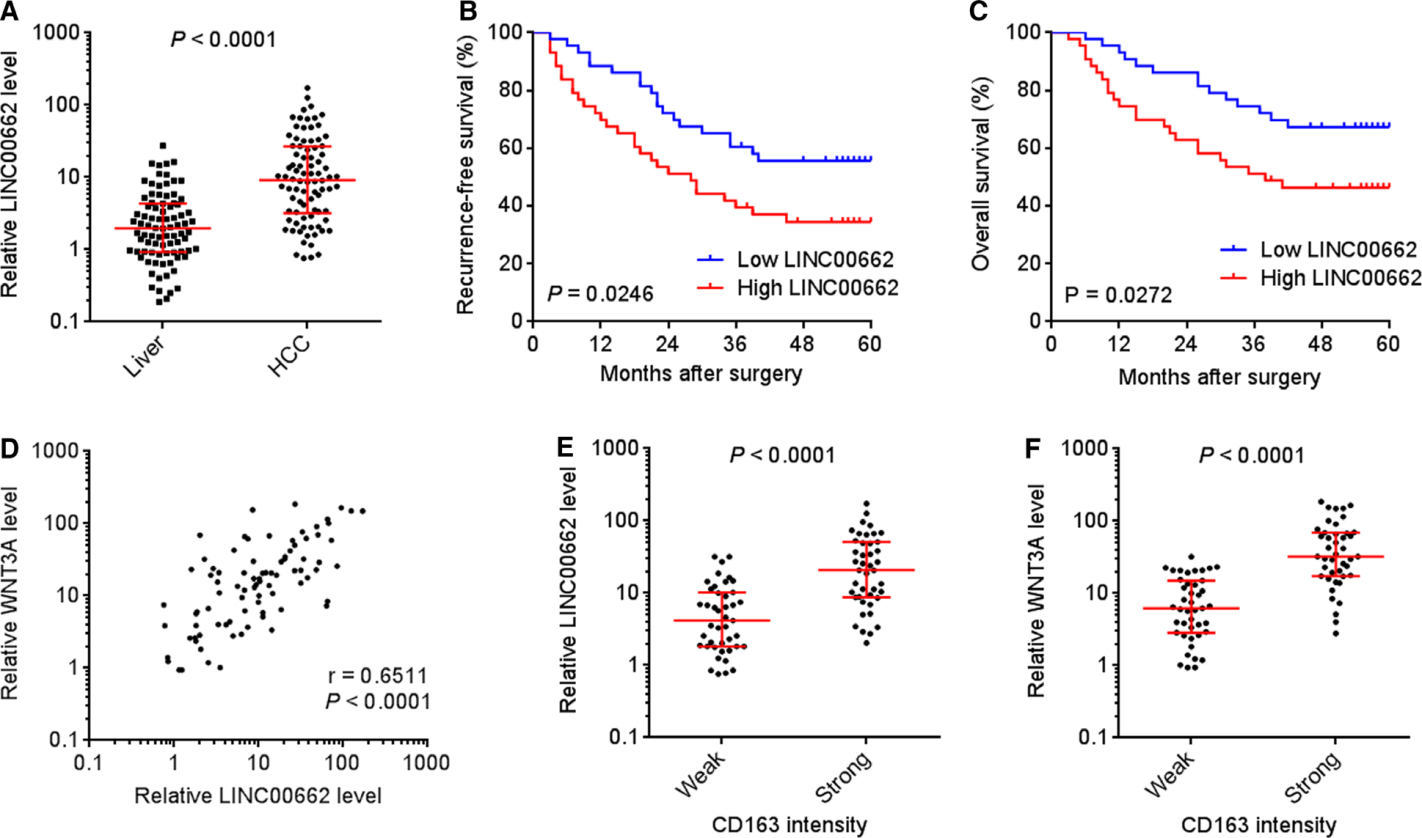
LINC00662 is increased in HCC tissues and associated with WNT3A expression, M2 macrophages polarization, and poor outcome of HCC patients. (A) LINC00662 expression in 86 pairs of HCC tissues and matched noncancerous liver tissues was measured by qRT/PCR. *P* < 0.0001 by Wilcoxon matched‐pairs signed rank test. (B, C) Kaplan–Meier survival analyses of the correlations between LINC00662 expression and recurrence‐free survival (B) or overall survival (C) of these 86 cases. The median expression level was used as cutoff. *P* values were calculated by log‐rank test. (D) The correlation between WNT3A and LINC00662 expression levels in these 86 HCC tissues. *r* = 0.6511, *P* < 0.0001 by Pearson correlation analysis. (E) LINC00662 expression levels in 86 HCC tissues with strong or weak M2 macrophage marker CD163 staining. (F) WNT3A expression levels in 86 HCC tissues with strong or weak M2 macrophage marker CD163 staining. The median CD163 staining intensity was used as the cutoff. *P* < 0.0001 by Mann–Whitney test.

To elucidate the clinical significances of LINC00662/WNT3A/M2 macrophage polarization regulatory axis in clinical tissues, we collected 86 pairs of HCC tissues and matched adjacent noncancerous liver tissues. Our data presented that LINC00662 was significantly increased in HCC tissues compared with adjacent noncancerous liver tissues (Fig. 7A). The correlation between LINC00662 expression and clinical characteristics in these 86 cases was analyzed. As presented in Table 1, high expression of LINC00662 was positively correlated with poor differentiation, large tumor size, and microvascular invasion. Furthermore, Kaplan–Meier survival analysis presented that high expression of LINC00662 was associated with poor recurrence‐free survival and overall survival (Fig. 7B,C). WNT3A expression in these 86 HCC tissues was further measured. Our data presented that the expression of WNT3A was markedly positively correlated with that of LINC00662 in HCC tissues (Fig. 7D). The HCC tissues with strong M2 macrophage marker CD163 staining showed higher expression of LINC00662 and WNT3A than that with weak CD163 staining (Fig. 7E,F and Fig. S4), which supporting the regulatory axis of LINC00662, WNT3A, and M2 macrophage polarization. Collectively, these data suggested that LINC00662 is increased in HCC tissues and associated with WNT3A expression, M2 macrophage polarization, and poor outcome of HCC patients.

## 4 Discussion

More than 80% of human genome transcribe RNAs. However, only about 2% of human genome encode proteins (Yan *et al.*, 2015). Thus, noncoding RNAs constitute important components of human transcriptome (Shah *et al.*, 2018). According to the length, two class of critical regulatory noncoding RNAs are gradually revealed (Riefolo *et al.*, 2019; Zhang *et al.*, 2017). lncRNAs are a class of noncoding RNAs with more than 200 nucleotides in length (Kopp and Mendell, 2018). miRNAs are another class of noncoding RNAs with 19–25 nucleotides in length (Yuan *et al.*, 2011). Aberrant expressions of lncRNAs and miRNAs have been found in many cancers, including HCC (Beltran‐Anaya *et al.*, 2019; Zhang *et al.*, 2013). Moreover, many lncRNAs and miRNAs also play important roles in various cancers, including HCC (Christensen *et al.*, 2016).

Recent advances have shown that tumor microenvironment (TME) also plays critical roles in tumor initiation and progression (Cassetta *et al.*, 2019; Liao *et al.*, 2019). The interaction between TME and cancer cells may promote the proliferation, metastasis, angiogenesis, and immune evasion of cancer cells (Carlson *et al.*, 2019; Salvagno *et al.*, 2019; Su *et al.*, 2019). However, the contributions of lncRNAs in the malignant interaction between TME and cancer cells are just beginning to be revealed (Huang *et al.*, 2018). lncRNA NKILA promotes cytotoxic T lymphocytes death via inhibiting NF‐κB activity and therefore promotes tumor immune evasion (Huang *et al.*, 2018). Except NF‐κB signaling, many other signaling pathways are also involved in the complex interactions between TME and cancer cells, such as Wnt/β‐catenin signaling and Notch signaling (Yang *et al.*, 2018; Ye *et al.*, 2019). Therefore, we hypothesized that the lncRNAs involved in these signaling pathways may further regulate the interactions between TME and cancer cells.

**Figure 8:**
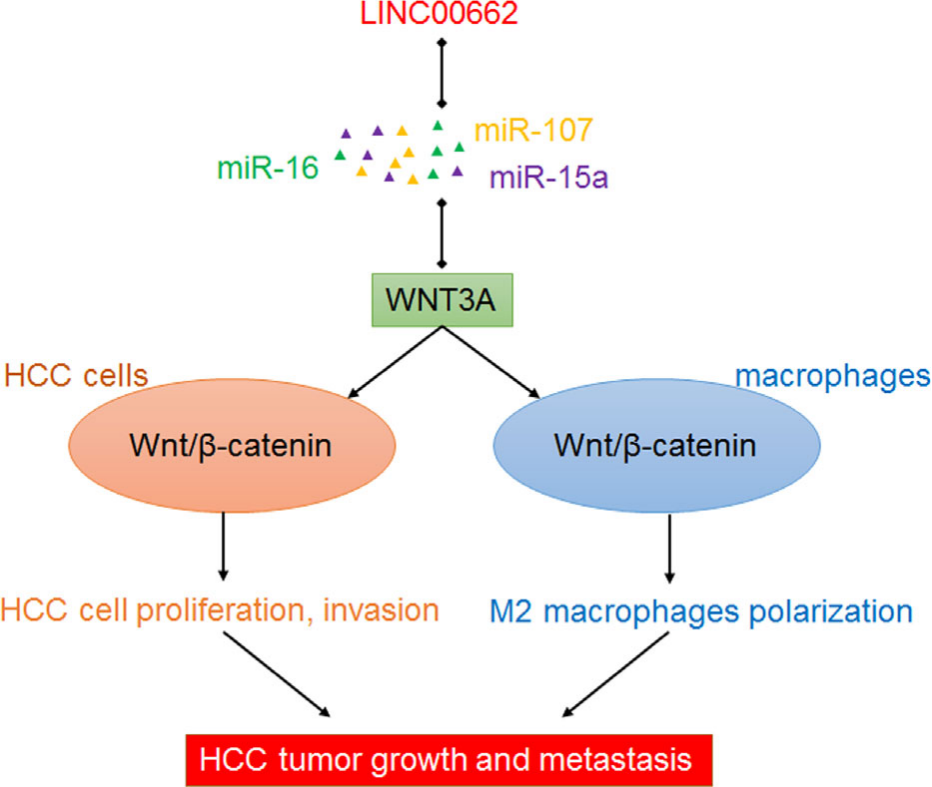
A schematic model of LINC00662 functions in HCC. LINC00662 upregulates WNT3A expression and secretion via competitively binding miR‐15a/16/107. Secreted WNT3A activates Wnt/β‐catenin signaling in HCC cells in an autocrine manner and further promotes HCC cell proliferation and invasion. On the other hand, secreted WNT3A activates Wnt/β‐catenin signaling in macrophages in a paracrine manner and further promotes M2 macrophages polarization. Finally, LINC00662 promotes HCC tumor growth and metastasis via upregulating WNT3A.

Combining bioinformation analyses and experimental verification, we found that lncRNA LINC00662 could physically bind miR‐15a, miR‐16, and miR‐107. These three miRNAs target WNT3A and repress Wnt/β‐catenin signaling. Via competitively binding miR‐15a/16/107, LINC00662 significantly upregulates WNT3A and further increases WNT3A secretion. Via inducing WNT3A secretion, LINC00662 activates Wnt/β‐catenin signaling in HCC cells in an autocrine manner. More importantly, via inducing WNT3A secretion, LINC00662 also activates Wnt/β‐catenin signaling in macrophages in a paracrine manner. Through activating Wnt/β‐catenin signaling in macrophages, LINC00662 further induces M2 macrophages polarization. The regulatory roles of LINC00662 on WNT3A, Wnt/β‐catenin signaling activation, and M2 macrophage polarization were verified in both HCC cell models *in vitro* and HCC xenograft growth and liver metastasis models *in vivo*. The correlations between LINC00662, WNT3A, M2 macrophage polarization were further found in clinical HCC tissues, which support the modulation of Wnt/β‐catenin signaling and M2 macrophage polarization by LINC00662 in human. Gain‐of‐function and loss‐of‐function assays showed that LINC00662 accelerates HCC cell proliferation cell cycle progression, and cell invasion, and inhibits HCC cell apoptosis. LINC00662 also promotes HCC xenograft growth and liver metastasis *in vivo*. The main findings of this study are presented in Fig. 8.

The gene coding LINC00662 is located in chromosome 19q11. LINC00662 has 2085 nucleotides in length and contains three exons. LINC00662 is mainly located in the cytoplasm and has comparable expression levels with miR‐15a/16/107, which support LINC00662 acting as a ceRNA of miR‐15a/16/107. Both TCGA data and our own clinical tissues presented that LINC00662 is highly expressed in HCC tissues compared with noncancerous liver tissues, and high expression of LINC00662 is associated with poor survival of HCC patients. Thus, this study identifies LINC00662 as an oncogenic lncRNA in HCC.

During our study, several studies about the expressions and roles of LINC00662 are reported. These reports showed that LINC00662 is highly expressed and plays oncogenic roles in acute myeloid leukemia, gastric cancer, prostate cancer, lung cancer, and oral squamous cell carcinoma via various mechanisms (Gong *et al.*, 2018; Li *et al.*, 2019; Liu *et al.*, 2019b; Liu *et al.*, 2018; Xu *et al.*, 2019). These studies combining with our findings together provide strong evidences to support LINC00662 as an important oncogenic lncRNA. The various functional mechanisms and oncogenic roles of LINC00662 further suggest the extensive involvements of LINC00662 in many aspects of cancers. The reasons underlying the various action mechanisms of LINC00662 in different cancers need further investigation.

## 5 Conclusion

In summary, this study finds that LINC00662 is upregulated in HCC and associated with tumor size, invasion, and poor survival of HCC patients. LINC00662 upregulates WNT3A expression and secretion via competitively binding miR‐15a, miR‐16, and miR‐107. Via upregulating WNT3A, LINC00662 activates Wnt/β‐catenin signaling in HCC cells in an autocrine manner and further promotes HCC cell proliferation, cell cycle, and invasion and represses HCC cell apoptosis. On the other hand, via upregulating WNT3A, LINC00662 activates Wnt/β‐catenin signaling in macrophages in a paracrine manner and further promotes M2 macrophages polarization. Therefore, LINC00662 significantly promotes HCC tumor growth and metastasis *in vivo*. Our findings suggest LINC00662 as a potential prognostic biomarker and therapeutic target for HCC.

## Conflict of interest

The authors declare no conflict of interest.

## Author contributions

DB and XT conceived and designed the project. XT, YW, YY, JW, and MN conducted the experiments and acquired the data. DB, XT, SG and TQ analyzed the data. DB and XT wrote the paper. All authors approved the final manuscript.

## Supporting information


**Fig. S1.** The expression and characters of LINC00662 in HCC.

**Fig. S2.** LINC00662 promotes HCC cell proliferation, cell cycle, and invasion, and represses cell apoptosis.

**Fig. S3.** LINC00662 upregulates WNT3A expression *in vivo*.

**Fig. S4.** Representative images of CD163 IHC staining in HCC tissues.

**Fig. S5.** Uncropped images of western blots.

**Table S1.** Primer sequences used in this study.
