# Supplementary figures and images for: Long noncoding RNA LINC00662 promotes M2 macrophage polarization and hepatocellular carcinoma progression via activating Wnt/β‐catenin signaling
Source: Mol Oncol. 2019 Dec 21;14(2):462–83. doi: 10.1002/1878-0261.12606 (PMC6998656; doi:10.1002/1878-0261.12606)

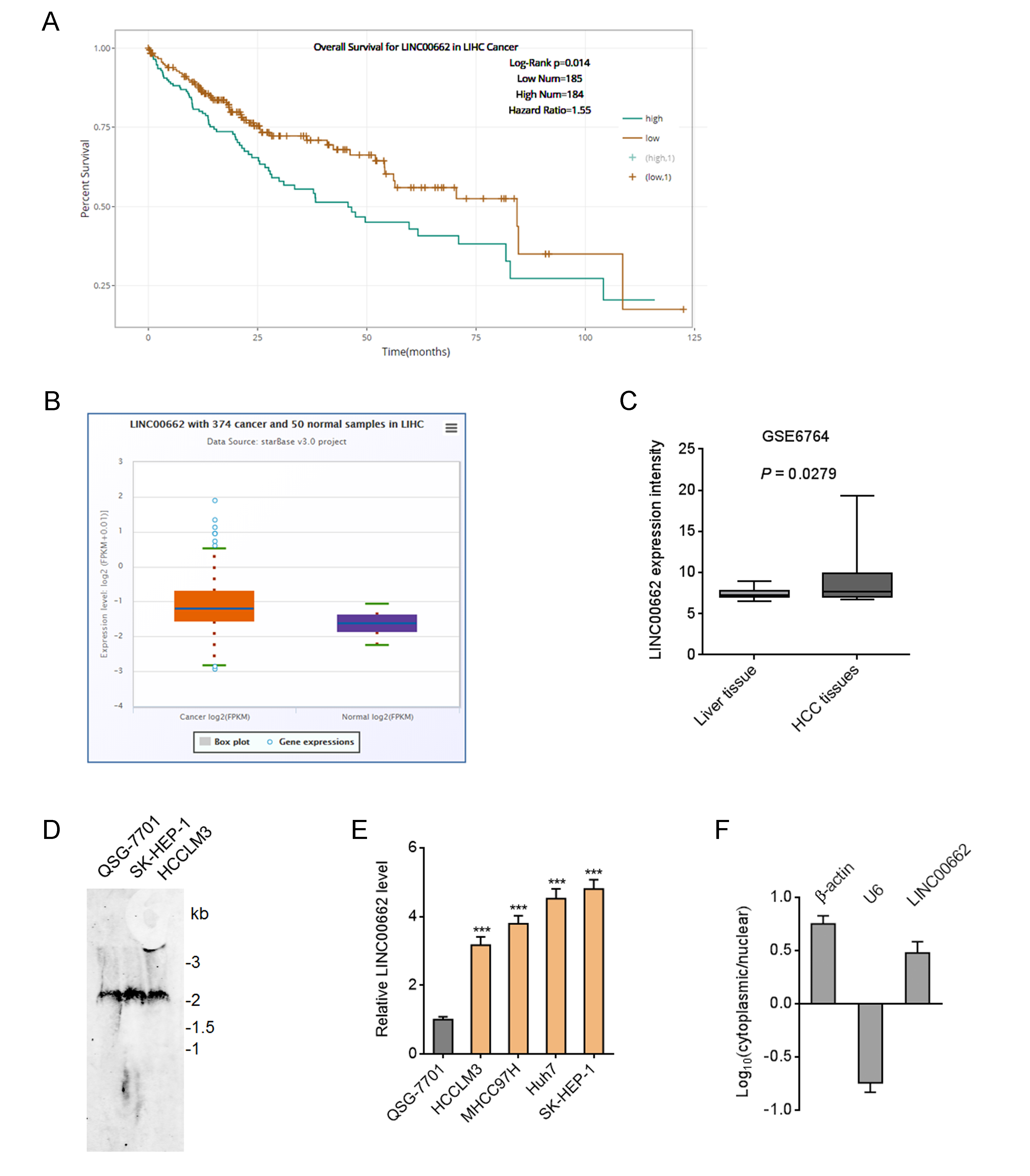

Supplement: Supplementary file 1 — Fig. S1. The expression and characters of LINC00662 in HCC. [file MOL2-14-462-s001.tif]

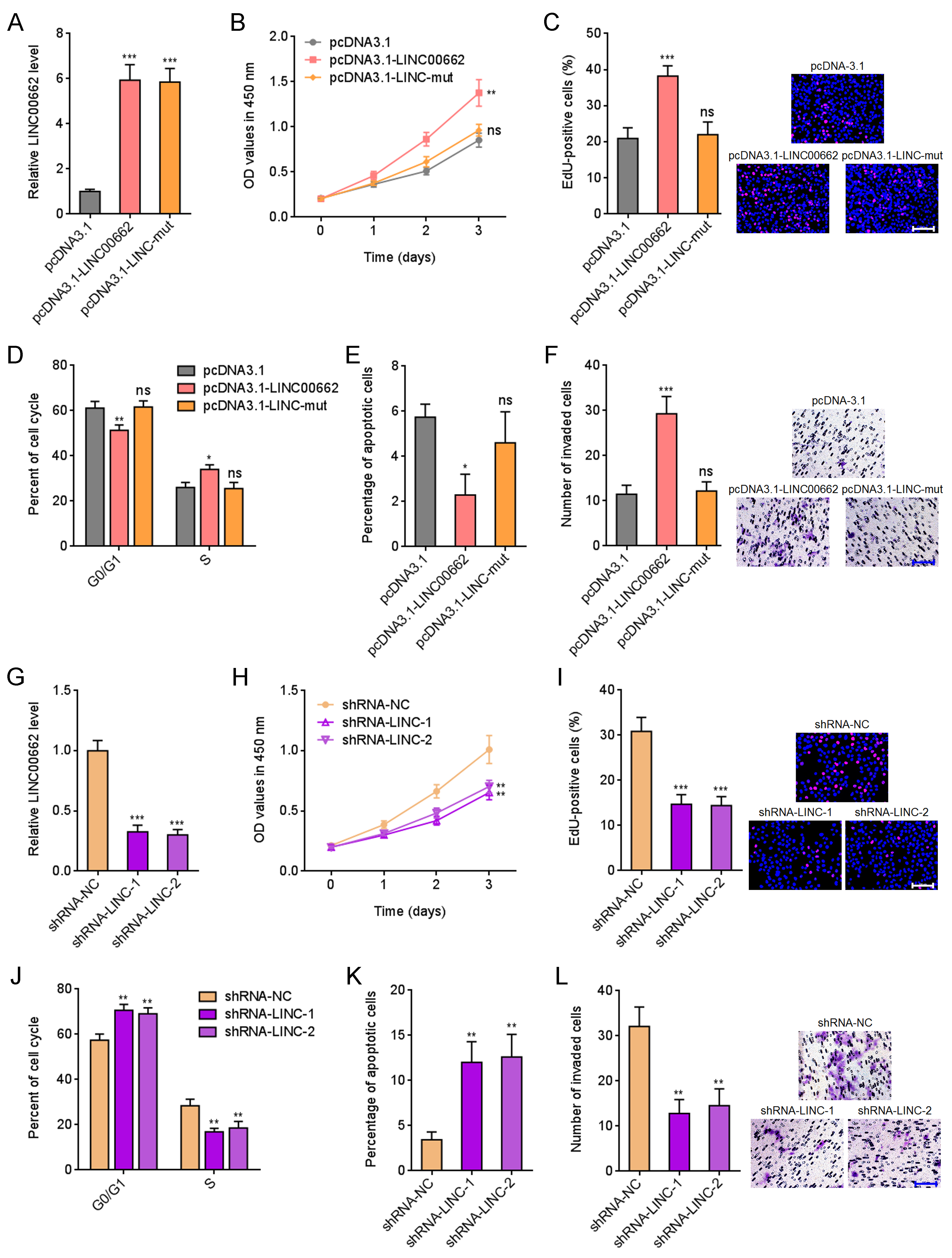

Supplement: Supplementary file 2 — Fig. S2. LINC00662 promotes HCC cell proliferation, cell cycle, and invasion, and represses cell apoptosis. [file MOL2-14-462-s002.tif]

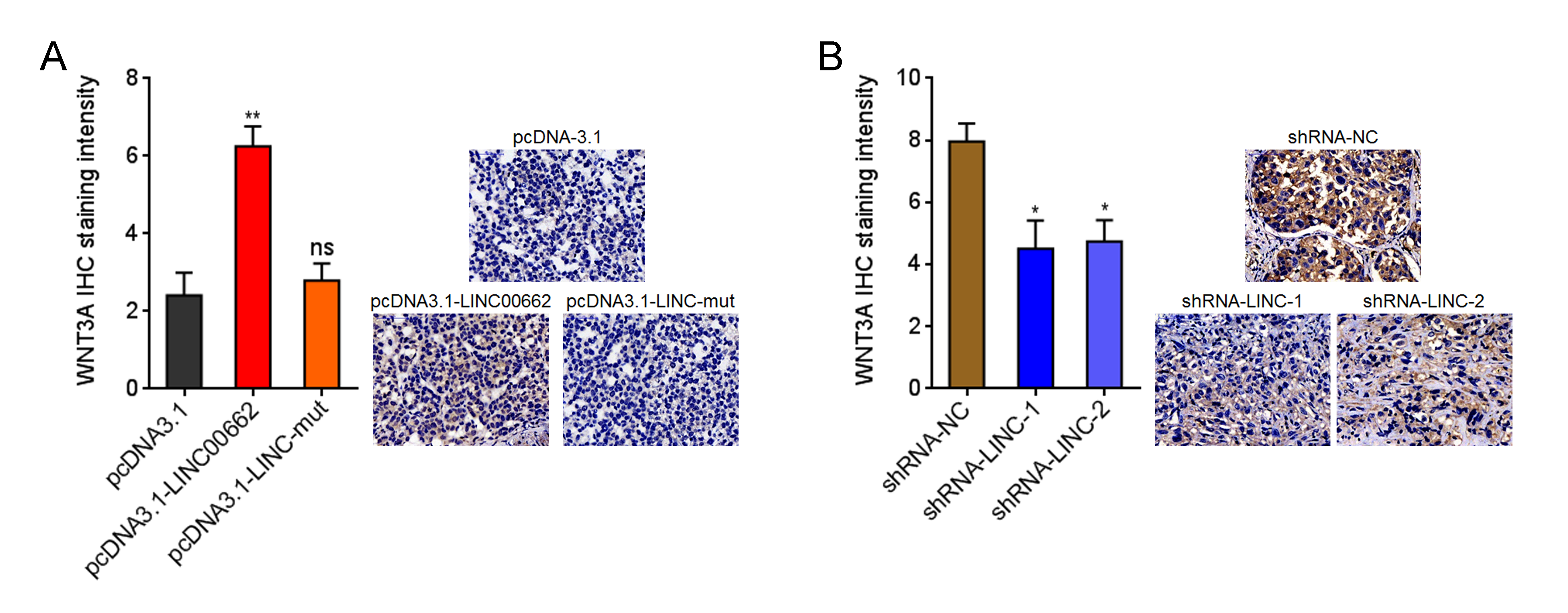

Supplement: Supplementary file 3 — Fig. S3. LINC00662 upregulates WNT3A expression in vivo. [file MOL2-14-462-s003.tif]

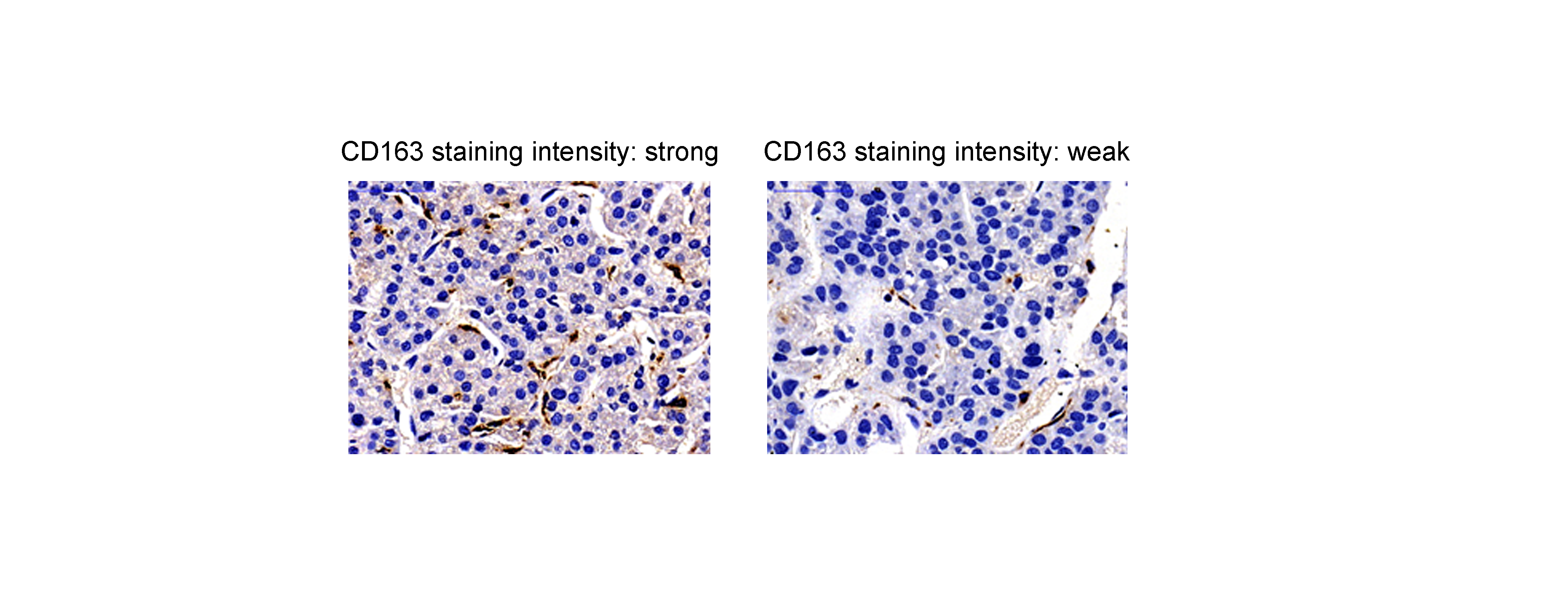

Supplement: Supplementary file 4 — Fig. S4. Representative images of CD163 IHC staining in HCC tissues. [file MOL2-14-462-s004.tif]

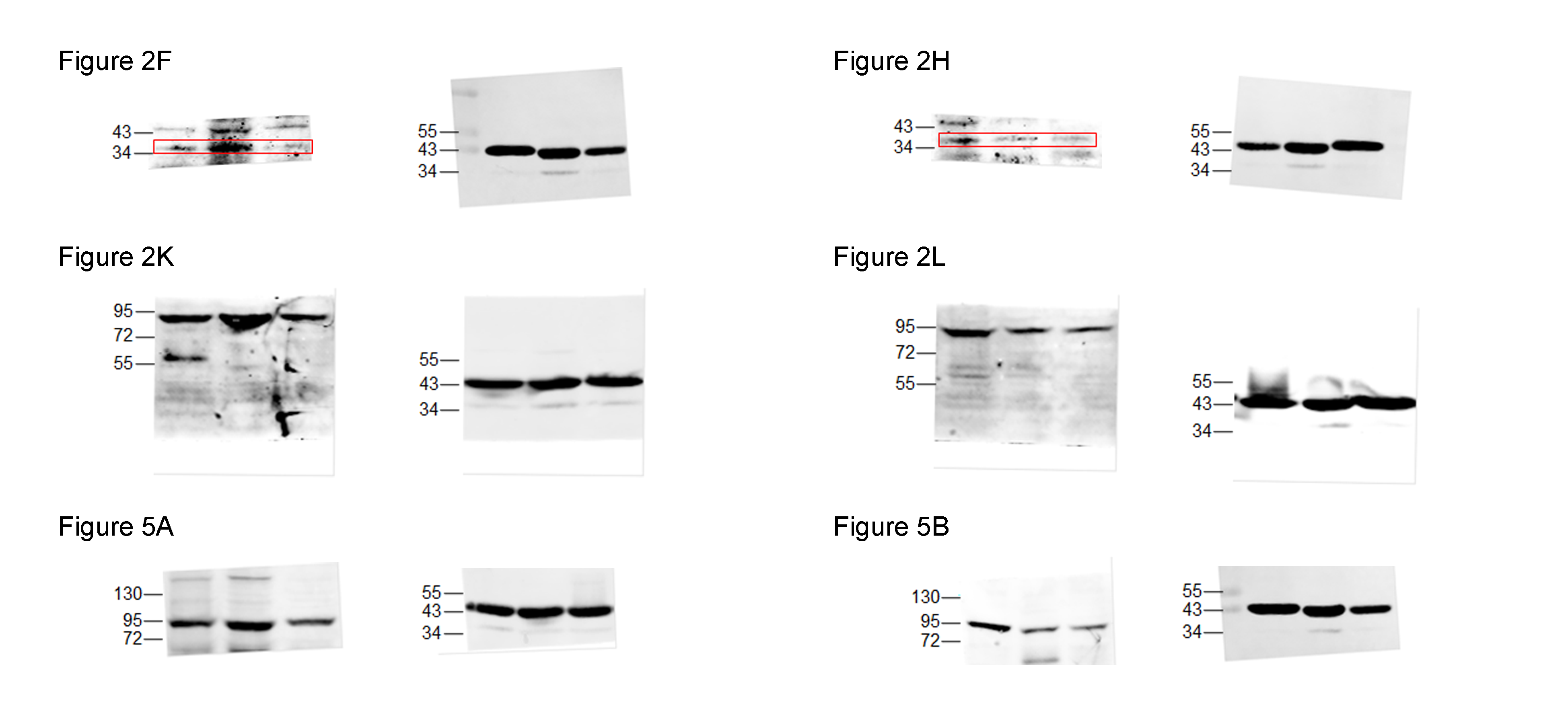

Supplement: Supplementary file 5 — Fig. S5. Uncropped images of western blots. [file MOL2-14-462-s005.tif]
