# Supplementary material for: Long noncoding RNA LINC00662 promotes M2 macrophage polarization and hepatocellular carcinoma progression via activating Wnt/β‐catenin signaling
Source: Mol Oncol. 2019 Dec 21;14(2):462–83. doi: 10.1002/1878-0261.12606 (PMC6998656; doi:10.1002/1878-0261.12606)
Supplement: Supplementary file 6 — Table S1. Primer sequences used in this study. [file MOL2-14-462-s006.docx]

**Supplementary Table 1.** Primer sequences used in this study.

| Targets | Name | Sequence |
| --- | --- | --- |
| **qRT-PCR primers** | | |
| LINC00662 | Sense | 5'-TGGACATCTGTCTGGAGG-3' |
|  | Anti-sense | 5'-GGCTGAGGCATAAGAATCG-3' |
| WNT3A | Sense | 5'-TGAACCGCCACAACAACGA-3' |
|  | Anti-sense | 5'-GCTTCTCCACCACCATCTC-3' |
| cyclin D1 | Sense | 5'-ACAACTTCCTGTCCTACTACC-3' |
|  | Anti-sense | 5'-TCCTCCTCCTCTTCCTCCT-3' |
| c-Myc | Sense | 5'-CTTCCCCTACCCTCTCAA-3' |
|  | Anti-sense | 5'-CGATTTCTTCCTCATCTTCT-3' |
| IL-12 | Sense | 5'-GAGGGGACAACAAGGAGT-3' |
|  | Anti-sense | 5'-TCAGGGAGAAGTAGGAATG-3' |
| iNOS | Sense | 5'-AGCCTGTGAGACGTTTGATG-3' |
|  | Anti-sense | 5'-GCTGGATGTCGGACTTTGT-3' |
| TNF-α | Sense | 5'-TCAGCCTCTTCTCCTTCCT-3' |
|  | Anti-sense | 5'-GGTTATCTCTCAGCTCCAC-3' |
| CD163 | Sense | 5'-AGCAGAGTTTGGTCAGGG-3' |
|  | Anti-sense | 5'-GGCTTTTTGTGGGGTTTTC-3' |
| IL-10 | Sense | 5'-GTGGAGCAGGTGAAGAAT-3' |
|  | Anti-sense | 5'-AAATGGGGGTTGAGGTATC-3' |
| ARG1 | Sense | 5'-GATGTGAAGGATTATGGGGA-3' |
|  | Anti-sense | 5'-GGTTGTCAGTGGAGTGTTG-3' |
| MRC1 | Sense | 5'-ACTGTTTTGGTTGGGATTG-3' |
|  | Anti-sense | 5'-TGGTGGATTGTCTTGAGG-3' |
| β-actin | Sense | 5'-GGGAAATCGTGCGTGACATTAAG-3' |
|  | Anti-sense | 5'-TGTGTTGGCGTACAGGTCTTTG-3' |
| U6 | Sense | 5'-GCTTCGGCAGCACATATACTAAAAT-3' |
|  | Anti-sense | 5'-CGCTTCACGAATTTGCGTGTCAT-3' |
| **Northern blot** | | |
| LINC00662 | Sense | 5'-CAGAATCTCCGTGGACAT-3' |
|  | Anti-sense | 5'-ACACTGGAACAGTATAAAGC-3' |
| **Vectors construction** | | |
| pcDNA3.1- LINC00662 | Sense | 5'-GGGGTACCGTAGTCCGGCCGCCCTGTGA-3' |
|  | Anti-sense | 5'-GGAATTCCACCACGACCAAGTGCAATTTATTC-3' |
| shRNA-NC | Sense | 5'-CACCGTTCTCCGAACGTGTCACGTCAAGAGATTACGTGACACGTTCGGAGAATTTTTTG-3' |
|  | Anti-sense | 5'-GATCCAAAAAATTCTCCGAACGTGTCACGTAATCTCTTGACGTGACACGTTCGGAGAAC-3' |
| shRNA-LINC-1 | Sense | 5'-CACCGCTGCTGCCACTGTAATAAAGTTCAAGAGACTTTATTACAGTGGCAGCAGCTTTTTTG-3' |
|  | Anti-sense | 5'-GATCCAAAAAAGCTGCTGCCACTGTAATAAAGTCTCTTGAACTTTATTACAGTGGCAGCAGC-3' |
| shRNA-LINC-2 | Sense | 5'-CACCGCTGCTACTATGCTGAATTTATTCAAGAGATAAATTCAGCATAGTAGCAGCTTTTTTG-3' |
|  | Anti-sense | 5'-GATCCAAAAAAGCTGCTACTATGCTGAATTTATCTCTTGAATAAATTCAGCATAGTAGCAGC-3' |
| pmirGLO- LINC00662 | Sense | 5'-CGAGCTCCACGCTTCTGAAACTGGT-3' |
|  | Anti-sense | 5'-CCCTCGAGAGGTGTCTGCTTTTGCTAT-3' |
| pmirGLO- WNT3A | Sense | 5'-CGAGCTCTTATGGTGGATGAGGCTTC TTC-3' |
|  | Anti-sense | 5'-CCCTCGAGCGTCTAACTCCGTTGGACAG-3' |
