# Supplementary material for: Long noncoding RNA LINC00662 promotes M2 macrophage polarization and hepatocellular carcinoma progression via activating Wnt/β‐catenin signaling
Source: Mol Oncol. 2019 Dec 21;14(2):462–83. doi: 10.1002/1878-0261.12606 (PMC6998656; doi:10.1002/1878-0261.12606)
Supplement: Supplementary file 7 [file MOL2-14-462-s007.docx]

**Supplementary figure legends**

**Fig. S1.** The expression and characters of LINC00662 in HCC. (A) Kaplan-Meier survival analyses of the correlation between LINC00662 expression and overall survival of HCC patients using TCGA data. *P* = 0.014 by log-rank test. (B) LINC00662 expression in 374 HCC tissues and 50 normal liver tissues from TCGA data. *P* < 0.0001 by Mann Whitney test. (C) LINC00662 (probe number 1558256_at) expression in 40 HCC tissues and 35 noncancerous liver tissues from GSE6764 data. *P* = 0.0279 by Mann Whitney test. (D) Northern blot analysis of LINC00662 in normal liver cell QSG-7701 and HCC cells SK-HEP-1 and HCCLM3. (E) The expression of LINC00662 in normal liver cell QSG-7701 and HCC cells HCCLM3, MHCC97H, Huh7, and SK-HEP-1 was measured by qRT-PCR. ****P* < 0.001 by one-way ANOVA followed by Dunnett's multiple comparisons test. (F) The levels of LINC00662 in purified cytoplasmic and nuclear RNAs were measured by qRT-PCR. β-actin and U6 serve as cytoplasmic and nuclear control, respectively.

**Fig. S2.** LINC00662 promotes HCC cell proliferation, cell cycle, and invasion, and represses cell apoptosis. (A) LINC00662 expression in wild-type or mutated LINC00662 stably overexpressed and control MHCC97H cells was measured by qRT-PCR. (B) Cell proliferation of wild-type or mutated LINC00662 stably overexpressed and control MHCC97H cells was detected by CCK-8 assays. OD values in 450 nm were collected to indicate cell proliferation. (C) Cell proliferation of wild-type or mutated LINC00662 stably overexpressed and control MHCC97H cells was detected by EdU incorporation experiments. The red colors indict EdU-positive nuclei and proliferative cells. Scale bars = 100 µm. (D) The cell numbers of wild-type or mutated LINC00662 stably overexpressed and control MHCC97H cells in G0/G1 and S phages were determined by PI staining and FACS. (E) Apoptosis of wild-type or mutated LINC00662 stably overexpressed and control MHCC97H cells was measured by Annexin V-PI staining and flow cytometry. (F) Invasion of wild-type or mutated LINC00662 stably overexpressed and control MHCC97H cells was measured by transwell invasion assays. Scale bars = 100 µm. (G) LINC00662 expression in LINC00662 stably silenced and control Huh7 cells was measured by qRT-PCR. (H) Cell proliferation of LINC00662 stably silenced and control Huh7 cells was detected by CCK-8 assays. OD values in 450 nm were collected to indicate cell proliferation. (I) Cell proliferation of LINC00662 stably silenced and control Huh7 cells was detected by EdU incorporation experiments. The red colors indict EdU-positive nuclei and proliferative cells. Scale bars = 100 µm. (J) The cell numbers of LINC00662 stably silenced and control Huh7 cells in G0/G1 and S phages were determined by PI staining and FACS. (K) Apoptosis of LINC00662 stably silenced and control Huh7 cells was measured by Annexin V-PI staining and flow cytometry. (L) Invasion of LINC00662 stably silenced and control Huh7 cells was measured by transwell invasion assays. Scale bars = 100 µm. Results are shown as mean ± standard error based on at least three independent experiments. **P* < 0.05, ***P* < 0.01, ****P* < 0.001, ns, not significant, by one-way ANOVA followed by Dunnett's multiple comparisons test.

**Fig. S3.** LINC00662 upregulates WNT3A expression in vivo. (A) WNT3A IHC staining of tumors derived from wild-type or mutated LINC00662 stably overexpressed and control HCCLM3 cells. (B) WNT3A IHC staining of tumors derived from LINC00662 stably silenced and control SK-HEP-1 cells.

**Fig. S4.** Representative images of CD163 IHC staining in HCC tissues.

**Fig. S5.** Uncropped images of western blots.
